# Supplementary material for: The Ciliopathy-Associated Cep104 Protein Interacts with Tubulin and Nek1 Kinase
Source: Structure. 2017 Jan 3;25(1):146–56. doi: 10.1016/j.str.2016.11.014 (PMC5222566; doi:10.1016/j.str.2016.11.014)
Supplement: Document S1. Figures S1–S6 and Tables S1 and S2 [file mmc1.pdf]

Structure, Volume *25*

Supplemental Information

The Ciliopathy-Associated Cep104 Protein Interacts with Tubulin and Nek1 Kinase

Caezar Al-Jassar, Antonina Andreeva, Deepak D. Barnabas, Stephen H. McLaughlin, Christopher M. Johnson, Minmin Yu, and Mark van Breugel

Table S1, related to Table 2

Human TOG Cep104$^{392\text{-}676}$ SeMet MAD dataset analysis.

Beamline	ID29 (ESRF)	
Space Group	P21	
Wavelength (Å)	0.97916 (peak)	0.97923 (inflection)
Unit Cell dimensions (Å)	a=51.8 b=53.3 c=154.4 α=90.0 β=91.8 γ=90.0	a=52.1 b=53.3 c=155.4 α=90.0 β=92.0 γ=90.0
Resolution (Å)	49.5 – 2.3	49.54 – 2.3
Completeness (overall / inner / outer shell)	99.6 / 99.1 / 99.5	99.5 / 98.7 / 99.5
Rmerge (overall / inner / outer shell)	0.145 / 0.045 / 1.050	0.127 / 0.034 / 0.987
Rpim (overall / inner / outer shell)	0.069 / 0.031 / 0.456	0.060 / 0.025 / 0.426
Mean I/σI (overall / inner / outer shell)	9.2 / 30.4 / 1.8	10.2 / 32.2 / 2.1
Multiplicity (overall / inner / outer shell)	6.9 / 6.5 / 7.1	6.9 / 6.5 / 7.1
Se sites found / expected	15 / 22 (16 SeMet residues are visible in the electron density map)	

Table S2, related to Table 2

Human ZNF Cep104$^{746\text{-}875}$ S763E Zn MAD dataset analysis.

Beamline	I04 (Diamond Light Source)		
Space Group	P212121		
Wavelength (Å)	1.28295 (peak)	1.28321 (inflection)	1.21984 (remote)
Unit Cell dimensions (Å)	a=73.9 b=80.5 c=118.3 α=90.0 β=90.0 γ=90.0	a=73.9 b=80.5 c=118.3 α=90.0 β=90.0 γ=90.0	a=73.9 b=80.5 c=118.3 α=90.0 β=90.0 γ=90.0
Resolution (Å)	80.5 – 2.2	80.5 – 2.2	80.5 – 2.2
Completeness (overall / inner / outer shell)	99.2 / 99.7 / 98.1	99.3 / 99.8 / 98.2	99.4 / 99.5 / 98.5
Rmerge (overall / inner / outer shell)	0.139 / 0.069 / 1.512	0.114 / 0.067 / 1.017	0.198 / 0.067 / 1.976
Rpim (overall / inner / outer shell)	0.046 / 0.024 / 0.510	0.038 / 0.025 / 0.342	0.065 / 0.024 / 0.657
Mean I/σI (overall / inner / outer shell)	11.3 / 30.5 / 1.9	12.0 / 28.3 / 2.5	9.6 / 28.4 / 1.8
Multiplicity (overall / inner / outer shell)	9.9 / 8.5 / 9.4	10.1 / 8.7 / 9.6	10.1 / 8.7 / 9.8
Zn found / expected	6 / 16		

Figure S1, related to Figure 1A

Homology model of the N-terminal APC10-like domain of Cep104.

A) Ribbon presentation of the N-terminal domain of Cep104 (rainbow-coloured from N- to C-terminus) together with an equivalent representation as molecular surface, coloured according to Consurf evolutionary conservation score from unconserved (cyan) to highly conserved (burgundy). **B)** Equivalent view onto the IFT25-IFT27 (pdb 2yc2) and the APC10-HSL1 (pdb 5g04) complexes. Note that IFT27 and HSL1 engage their corresponding APC10(-like) domain at a region that is conserved in the N-terminal, APC10-like domain in Cep104.

Figure S2, related to Figure 1B-D

Multiple sequence alignments and structural analyses of Cep104's TOG domain.

A) A structure-based, multiple sequence alignment of Cep104 TOG and other TOG domains with experimentally determined high-resolution structures (pdb 4ffb Stu2 TOG1, pdb 4g3a Mast/Orbit TOG1, pdb 2qk1 Stu2 TOG2, pdb 2qk2 Msps TOG2, pdb 4qmj ch-TOG TOG4, pdb 2of3 Zyg9 TOG3, pdb 3woy CLASP2 TOG2. Residues that were mutated in this study are marked with green circles. Structurally equivalent residues are shown in upper case. **B)** Multiple sequence alignment of Cep104 TOG of closely related homologs. Residues that were mutated in this study are marked with green circles. **C)** The tubulin-binding interface of Cep104 TOG is conserved and conformationally similar to other tubulin binding TOG domains. Globally fitted overlay of the ribbon representation of the Cep104 TOG structure with other TOG domains with experimentally determined high-resolution structures. Helices are displayed as cylinders. The top view onto the tubulin-binding interface reveals similar positions of the α-helices and their connecting loops, while that is not observed when the domain is viewed from the bottom. Used PDB codes were: 4ffb (Stu2 TOG1), 2of3 (Zyg9 TOG3), 2qk1 (Stu2 TOG2), 2qk2 (Msps TOG2), 3woy (CLASP2 TOG2), 4g3a (Mast/Orbit TOG1), 4qmj (ch-TOG TOG4). **D)** Cep104 TOG structure displayed as molecular surface coloured according to Consurf evolutionary conservation score from unconserved (cyan) to highly conserved (burgundy). View onto the tubulin-binding interface (left) or onto the bottom of the TOG domain (right).

Figure S3, related to Figure 2A

Candidates identified as putative Cep104 (proximity) interactors in BioID and cross-linking experiments.

A) Top, Coomassie stained SDS-PAGE gel showing the elutions from a Cep104 pull-down (employing site-specific biotinylation of Avitag-WT BirA tagged Cep104 in vivo) in the presence or absence of cross-linking. The gel corresponds to ~10% of the material that was subjected to mass-spectrometric analysis. Bottom, Proteins identified by mass-spectroscopy that showed a ten-fold or higher enrichment of exclusive unique peptide counts compared to the control. **B)** Top, Coomassie stained SDS-PAGE gel showing ~10% of the elution from a Cep104 BioID experiment (biotinylation of proteins in the vicinity of BirA* tagged Cep104 in vivo). The remainder of the elution was subjected to mass-spectrometric analysis. Bottom, proteins with a ten-fold or higher enrichment of exclusive unique peptide counts compared to the control.

Figure S4, related to Figure 2B

The Cep104 ZNF domain is necessary for the Cep104-Nek1 interaction in vivo.

Representative fluorescence micrographs of a re-routing and capture assay (Wong and

Munro, 2014) in U2OS cells with Nek1 and Cep104 constructs. N-terminally mCherry-tagged Cep104 or Cep104ΔZNF (Cep104$^{\Delta 746\text{-}880}$), fused at their C-terminus to the HA tag and the outer-mitochondrial membrane targeting transmembrane domain of monoamine oxidase (MAO) were co-transfected with GFP-Nek1 into U2OS cells, fixed after 24 hours and visualized using a confocal microscope. A mitochondrial stain (MitoTracker Deep-red, ThermoFisher Scientific) and DAPI were used to mark mitochondria and DNA respectively. Please note that ectopically expressed GFP-Nek1 reroutes only to the mitochondrially anchored Cep104 when Cep104's ZNF domain is present. Scale bars, 5 μm.

Figure S5, related to Figure 3A

Binding of CP110$^{906\text{-}936}$ and Nek1 to the Cep104 ZNF array is mutually exclusive. Right: Western blot showing a pull-down experiment with GST-Cep104 ZNF beads and lysates from Hek293 cells transiently overexpressing 3xFlag-tagged human Nek1 in the presence of an excess of recombinant CP110$^{906\text{-}936}$ or *Danio rerio* STIL$^{404\text{-}448}$. Please note that *Danio rerio* STIL$^{404\text{-}448}$ did not transfer/Ponceau-stain well as revealed by the Coomassie-stained SDS-PAGE gel of the input material shown on the left.

Figure S6, related to Figure 4

Multiple sequence alignment and structural comparison of Cep104's ZNF domain.

A) Marked with coloured lines and circles are the zinc-coordinating residues and the residues that constitute the three hydrophobic clusters that mediate the packing of the individual ZNFs into a globular domain. Indicated in red is residue S763 that was mutated (S763E) to increase solubility of the ZNF domain. **B)** The globular arrangement of the Cep104 ZNF array is novel. Comparison of the Cep104 ZNF domain with a similar ZNF array from TRAF6 (ribbon presentation, pdb 3hcs).

Supplementary References

Wong, M., and Munro, S. (2014). Membrane trafficking. The specificity of vesicle traffic to the Golgi is encoded in the golgin coiled-coil proteins. Science (New York, NY) *346*, 1256898.

Figure S1, related to Figure 1A

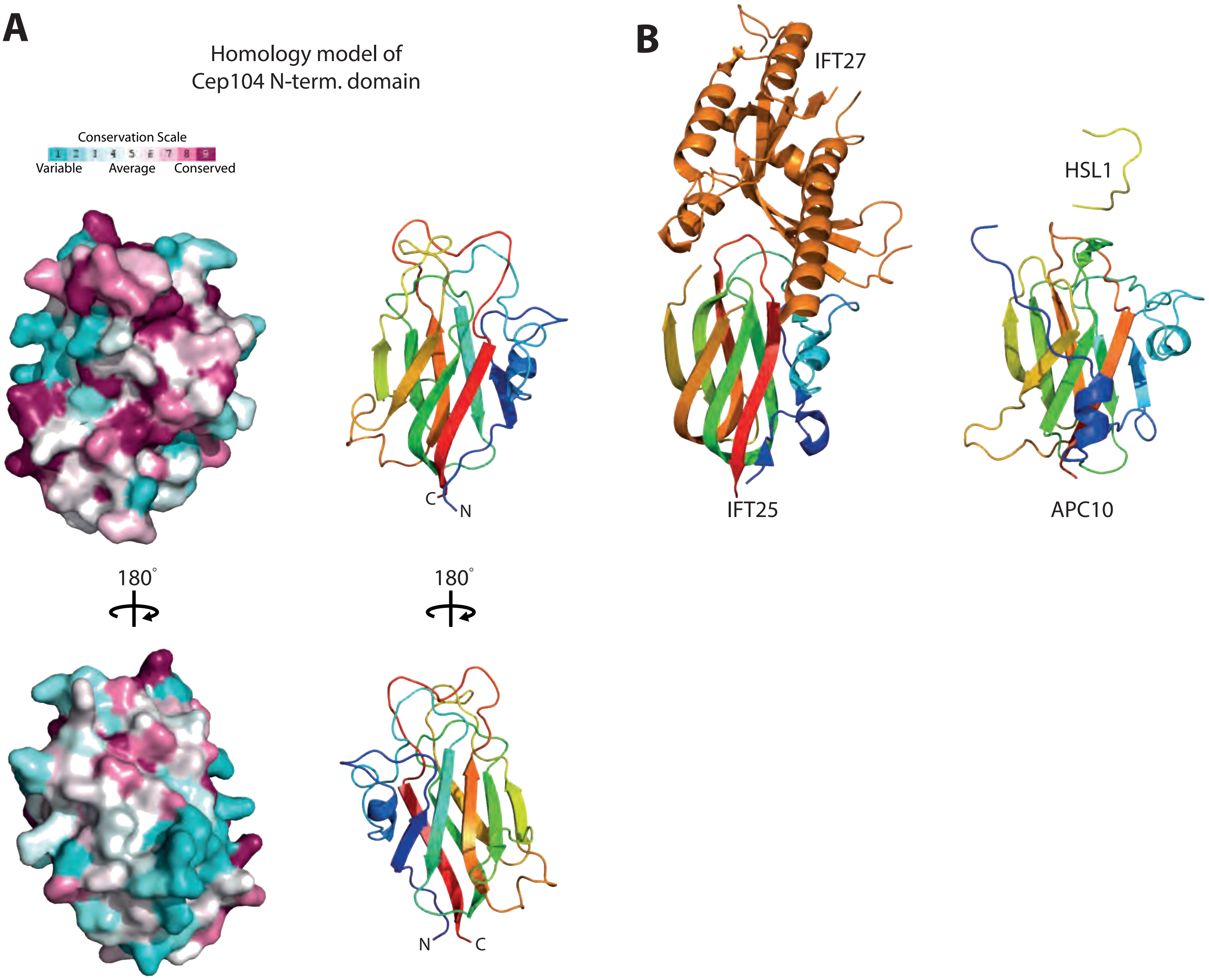

Figure S2, related to Figure 1B-D

A

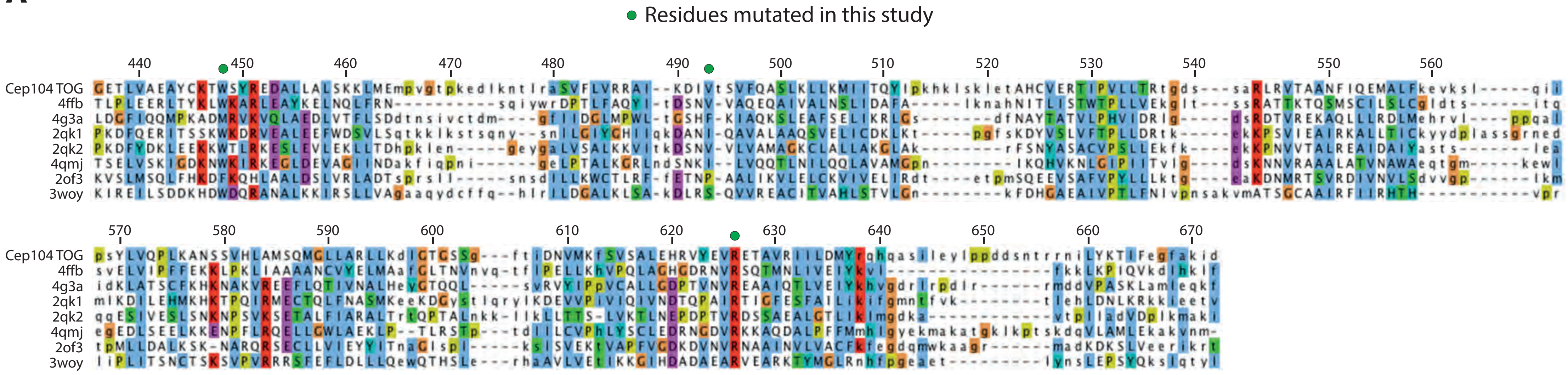

Figure S2, related to Figure 1B-D

B

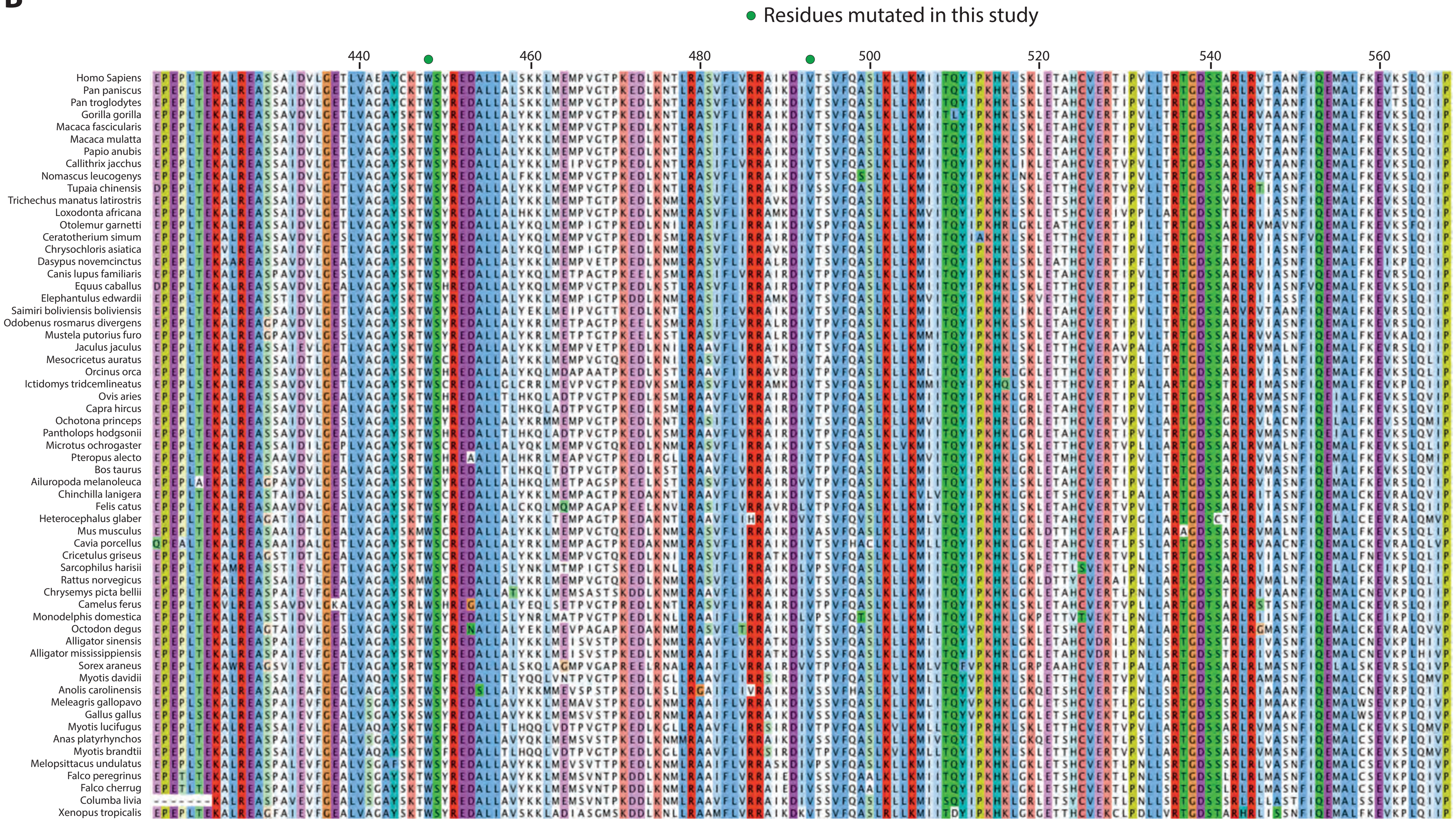

Figure S2, related to Figure 1B-D

B (continued)

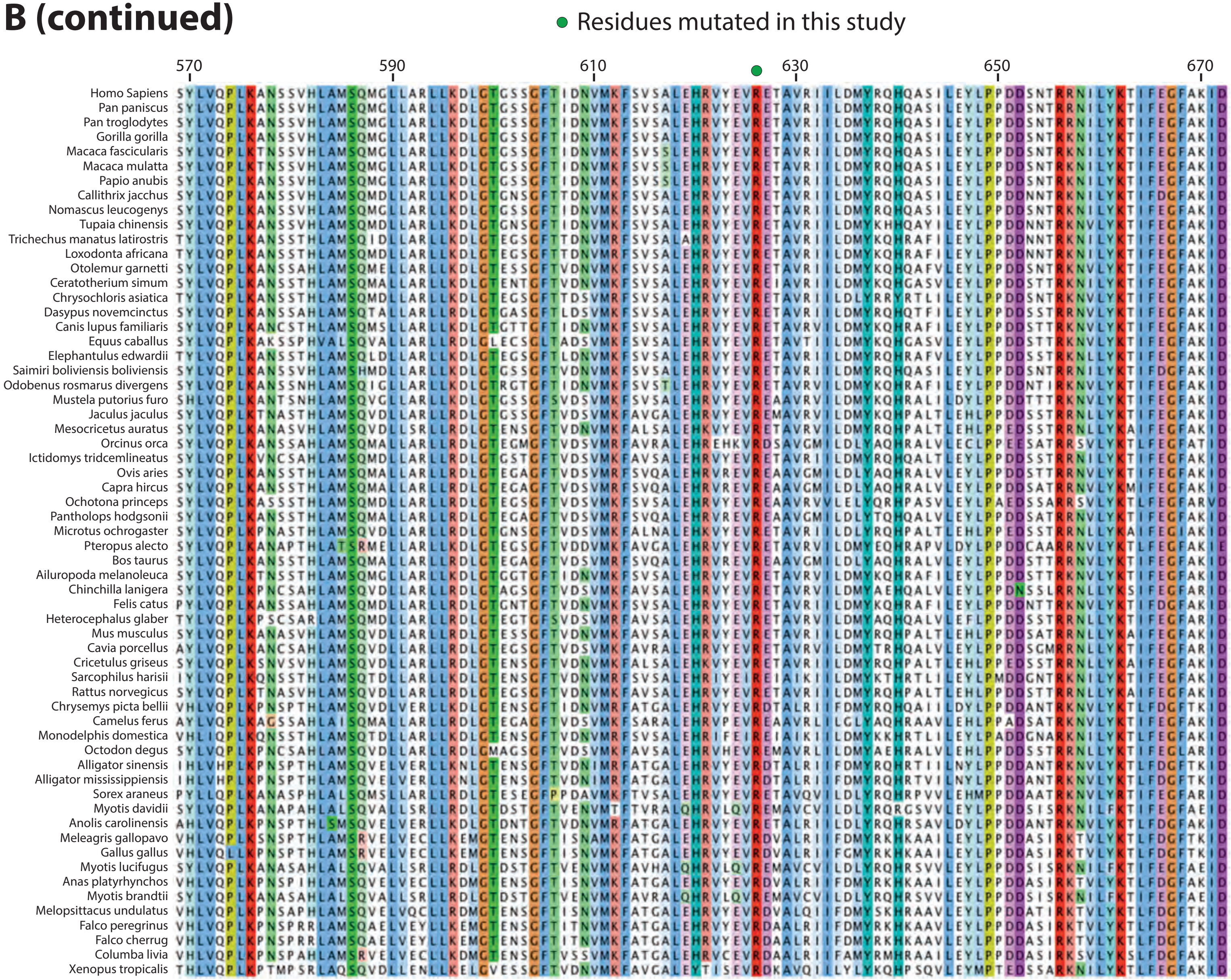

Figure S2, related to Figure 1B-D

C

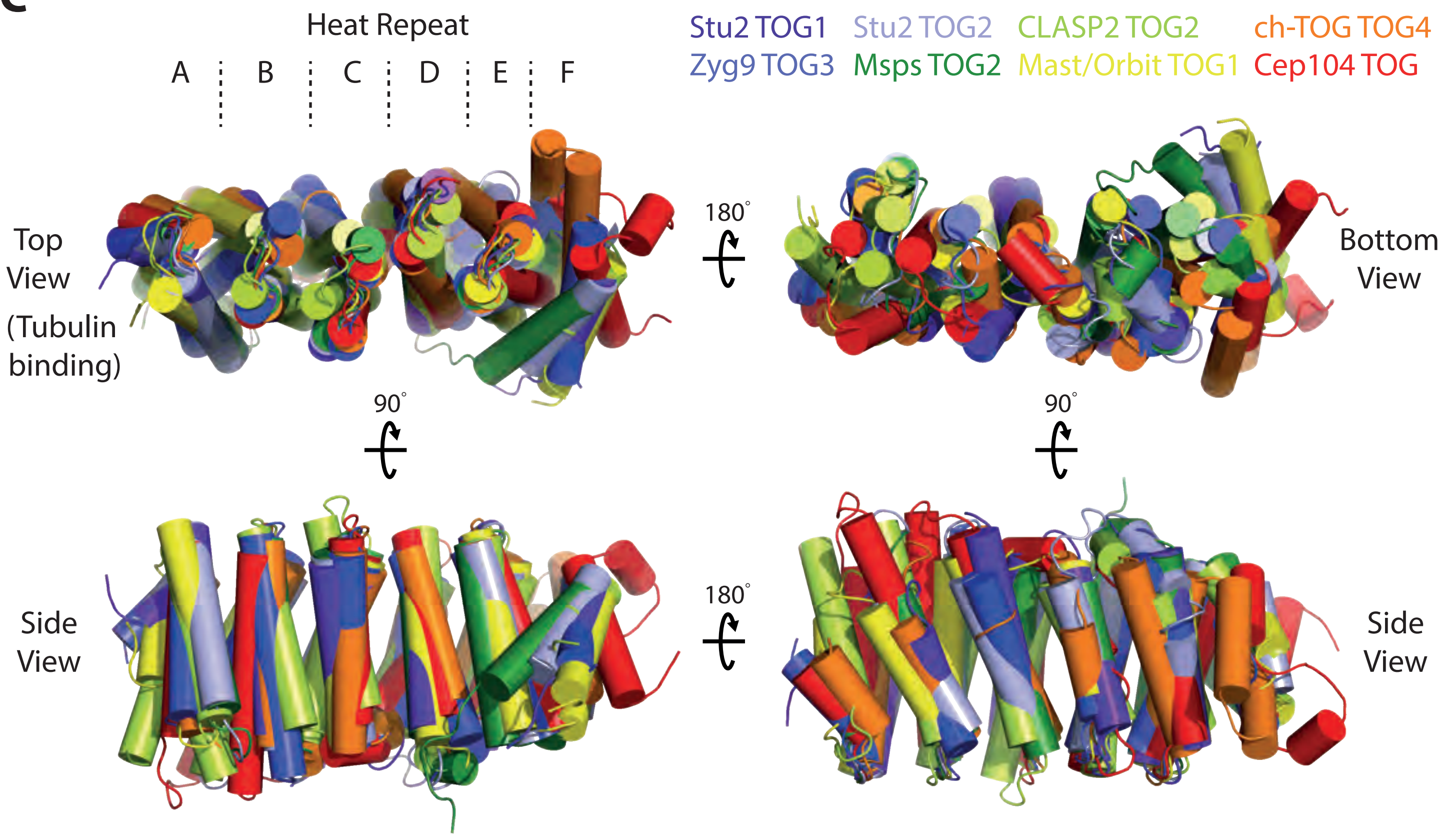

D

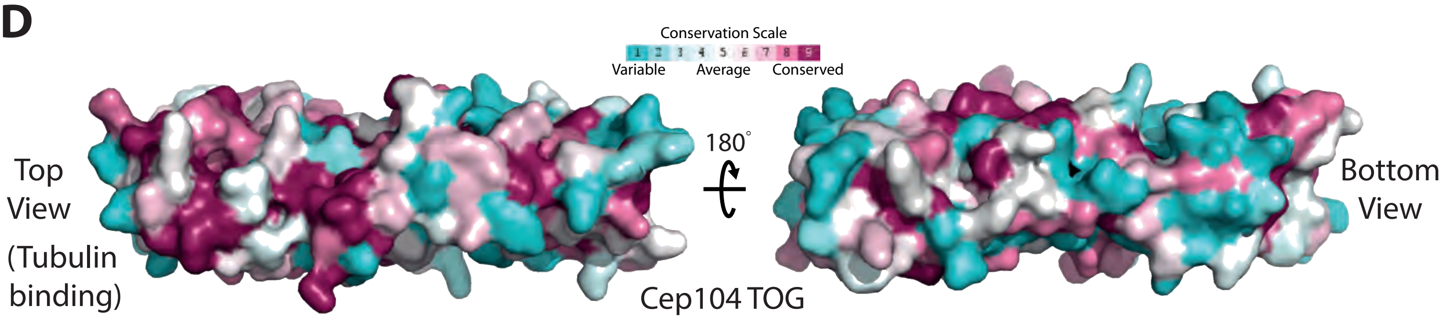

Figure S3, related to Figure 2A

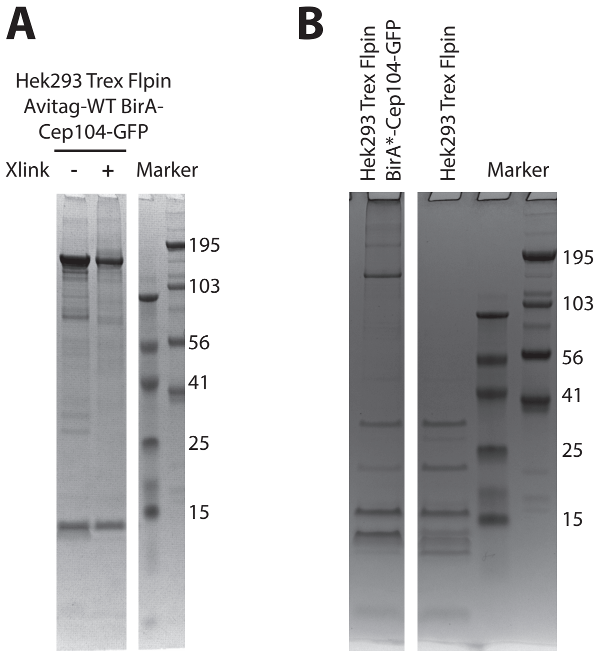

Xlinking

PCCB
LMNB1
MDH2
RBMX
RUVBL2
CLTC
MYH10
NCL
DNAJA2
B4E1G2
lmna
SFPQ
TPR
CCT2
ACTN4
EIF4G1
SPTAN1
B2R7C5
B2R8R5
Cep290
CSE1L
EIF3A
VCP
HUWE1
TNPO1
Nek1

BioID

HMPREF9541_01744	NUDC	lacZ	TCHP	NEDD1	SLK	PPP6R3
CEP104	PDLIM1	UBAP2L	ATXN2L	ZYX	TJP2	SRP68
FLNA	HSP90AB1	NUFIP2	TUBB	PA2G4	SMG7	PSMC2
CCT8	MAP4	Q53GZ6_HUMAN	PCBP1	EIF4G2	TRIM9	NOTCH2
AHNAK	EIF5	SNAP29	CAMSAP1	SPAG9	RASSF8	DBN1
PCM1	VIM	KIAA1009	DPYSL3	CSDE1	XIAP	SCIN
CEP170	AZI1	RANBP3	GEMIN5	RPAP3	CCDC66	SEC16A
CD2AP	MYH9	GPATCH1	B3KY28_HUMAN	PIBF1	IFT74	PSMD12
Q53HG7_HUMAN	NUP214	ANKRD17	SUGT1	TACC1	EEF2	CEP290
CCP110	RANGAP1	RUVBL1	CLINT1	TMF1	FKBP4	TCP1
CEP350	MRE11A	DDX3X	EPB41	DST	SYAP1	KTN1
EIF4G1	MPHOSPH9	DPYSL2	TNRC6B	RANBP2	CCT2	
MKL2	CSPP1	UBAP2	NSFL1C	HNRPK	EPB41L2	
TAGLN2	MAP7D3	DBNL	PKM	CCDC85C	RAI14	
HSPA1B	SKA3	KIAA1671	WWOX	EIF2A	B0FTY2_HUMAN	
ANKHD1	HAUS6	TP53BP2	A8KAL2_HUMAN	GOLGB1	KIAA1524	
CRKL	MAP1B	IRAK1	ANXA2	PLS3	ZC3H15	
LUZP1	TLN1	EIF4B	CKAP2	ENO1	WDR44	
LARP1	NEK1	HDLBP	DLGAP5	STIP1	B4DTX5_HUMAN	
SERBP1	TJP1	IRS4	DNAJB1	PXN	FAM21A	
CRK	ALMS1	ERC1	KNS2	VCL	EIF4ENIF1	
CEP97	CORO1B	CALD1	CENPH	SPAG5	EPS15	
PRRC2C	CNN3	C1orf198	BRAP	PPP1R12A	DNAJC7	
EPS15L1	DVL2	STAU1	CKAP5	KIF5B	REPS1	
MLLT4	EFHD2	VCPIP1	USP15	SNX1	CLMN	
OFD1	PDLIM5	PRRC2A	ABLIM1	ERBB2IP	SLAIN2	

Hits with ≥ 10-fold enrichment of exclusive, unique peptide counts compared to control

Figure S4, related to Figure 2B

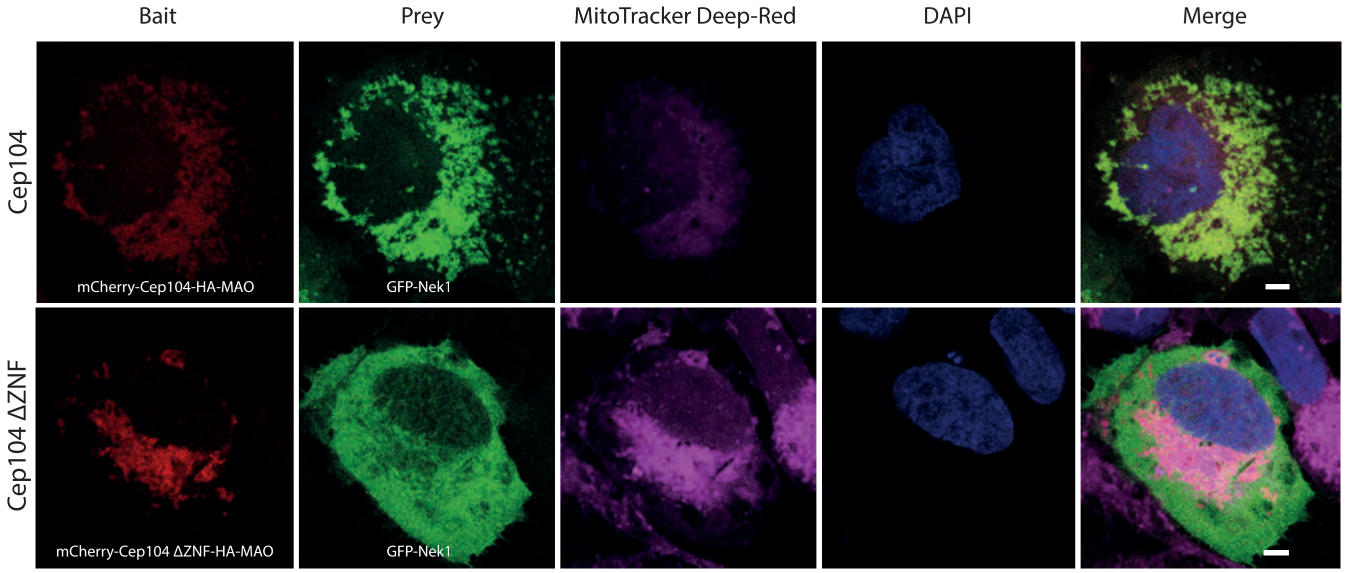

Figure S5, related to Figure 3A

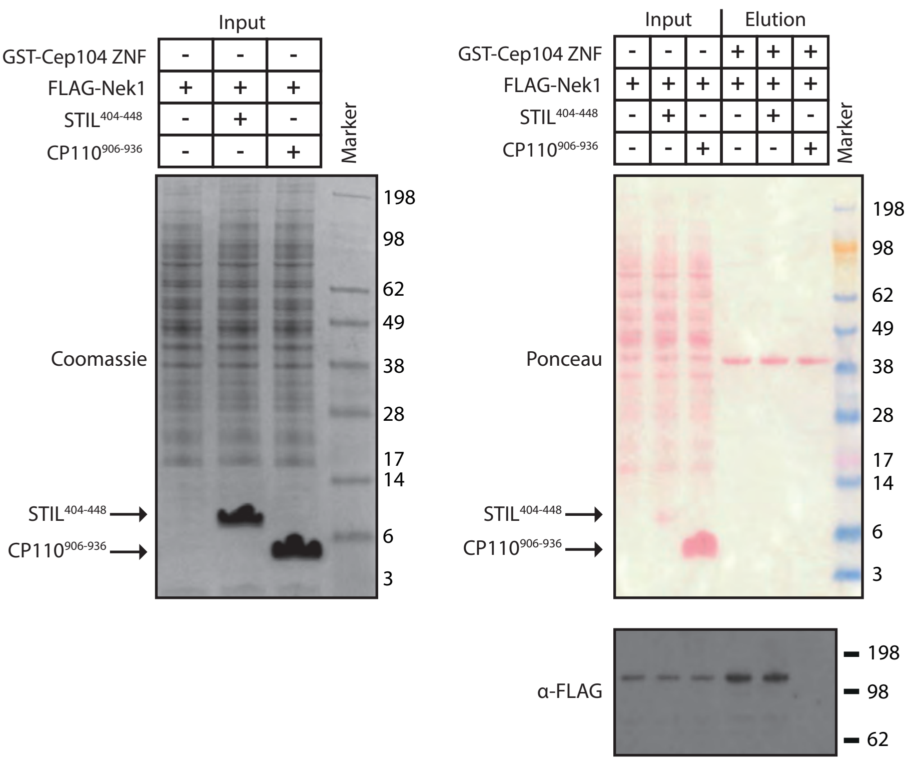

Figure S6, related to Figure 4

A

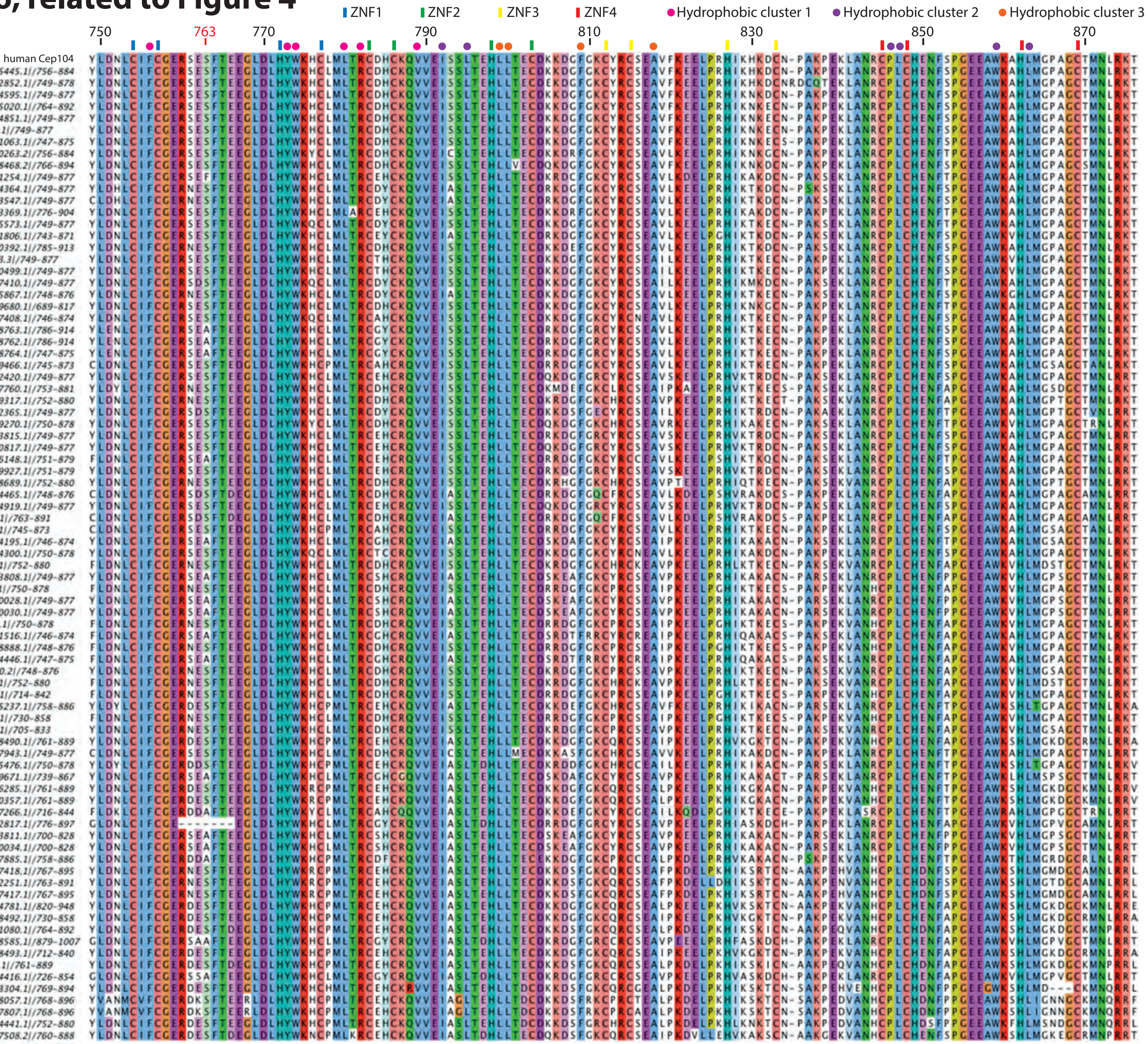

Figure S6, related to Figure 4

B

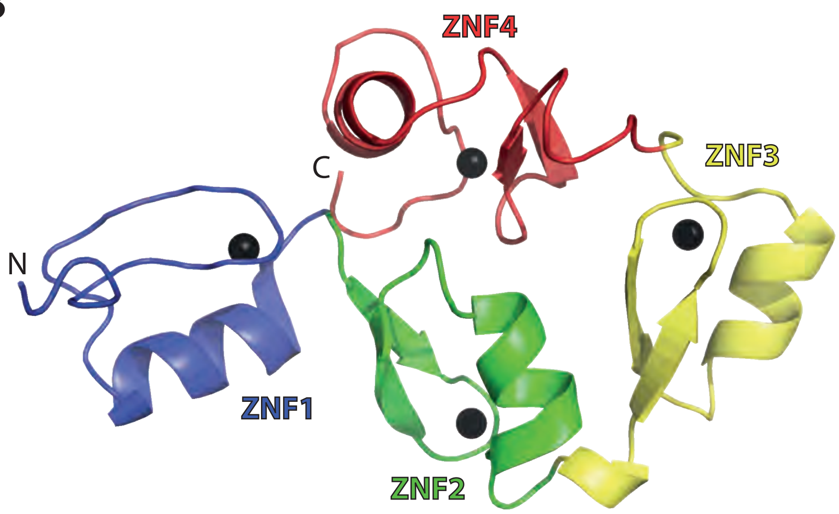

Cep104 ZNF array

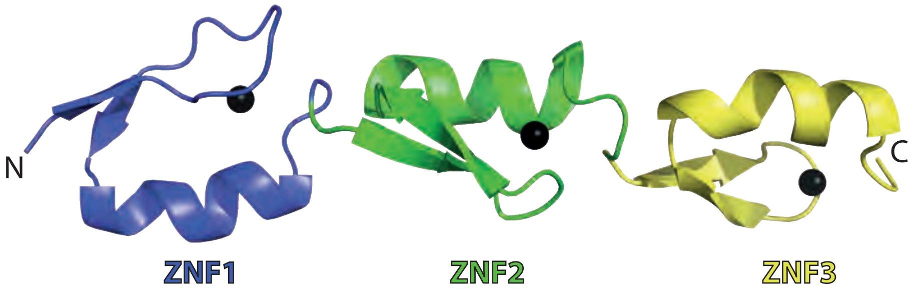

TRAF6 ZNF array
