# Supplementary material for: The Ciliopathy-Associated Cep104 Protein Interacts with Tubulin and Nek1 Kinase
Source: Structure. 2017 Jan 3;25(1):146–56. doi: 10.1016/j.str.2016.11.014 (PMC5222566; doi:10.1016/j.str.2016.11.014)
Supplement: Document S2. Article plus Supplemental Information [file mmc2.pdf]

Structure

Article

The Ciliopathy-Associated Cep104 Protein Interacts with Tubulin and Nek1 Kinase

Graphical Abstract

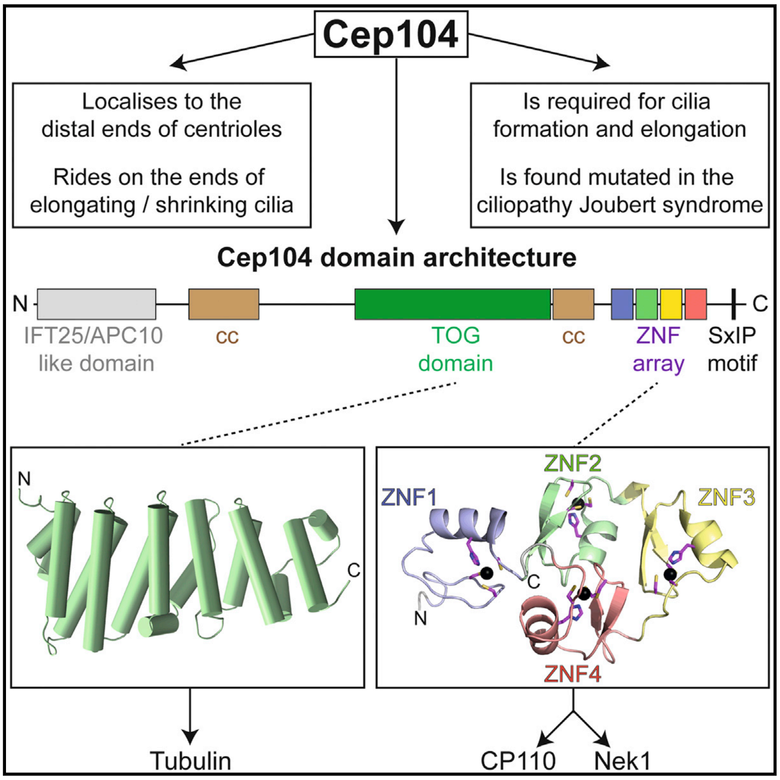

Authors

Caezar Al-Jassar, Antonina Andreeva, Deepak D. Barnabas, Stephen H. McLaughlin, Christopher M. Johnson, Minmin Yu, Mark van Breugel

Correspondence

vanbreug@mrc-lmb.cam.ac.uk

In Brief

Cep104 is essential for cilia formation. Al-Jassar et al. provide insights into Cep104's function by showing that it contains a tubulin-binding TOG domain. The authors also found that the ciliopathy-associated Nek1 kinase binds to Cep104 and that the centriole-capping protein CP110 competes for this binding.

Highlights

- The centriolar protein Cep104 contains a tubulin-binding TOG domain
- Nek1 kinase binds to a zinc finger array in Cep104
- Nek1 and the centriole-capping protein CP110 compete for binding to Cep104
- The Cep104's zinc finger array represents a novel arrangement of C2HC zinc fingers

Al-Jassar et al., 2017, Structure *25*, 146–156
January 3, 2017 © 2016 MRC Laboratory of Molecular Biology. Published by Elsevier Ltd.
http://dx.doi.org/10.1016/j.str.2016.11.014

Structure
Article

The Ciliopathy-Associated Cep104 Protein Interacts with Tubulin and Nek1 Kinase

Caezar Al-Jassar,[1,2] Antonina Andreeva,[1,2] Deepak D. Barnabas,[1] Stephen H. McLaughlin,[1] Christopher M. Johnson,[1] Minmin Yu,[1] and Mark van Breugel[1,3,*]
[1]Medical Research Council Laboratory of Molecular Biology, Francis Crick Avenue, Cambridge CB2 0QH, UK
[2]Co-first author
[3]Lead Contact
*Correspondence: vanbreug@mrc-lmb.cam.ac.uk
http://dx.doi.org/10.1016/j.str.2016.11.014

SUMMARY

Cilia are thin cell projections with essential roles in cell motility, fluid movement, sensing, and signaling. They are templated from centrioles that dock against the plasma membrane and subsequently extend their peripheral microtubule array. The molecular mechanisms underpinning cilia assembly are incompletely understood. Cep104 is a key factor involved in cilia formation and length regulation that rides on the ends of elongating and shrinking cilia. It is mutated in Joubert syndrome, a genetically heterogeneous ciliopathy. Here we provide structural and biochemical data that Cep104 contains a tubulin-binding TOG (tumor overexpressed gene) domain and a novel C2HC zinc finger array. Furthermore, we identify the kinase Nek1, another ciliopathy-associated protein, as a potential binding partner of this array. Finally, we show that Nek1 competes for binding to Cep104 with the distal centriole-capping protein CP110. Our data suggest a model for Cep104 activity during ciliogenesis and provide a novel link between Cep104 and Nek1.

INTRODUCTION

Cilia are hair-like projections present on the cell's plasma membrane. They are templated by centrioles, which, during ciliogenesis, dock against the cell membrane and subsequently extend their peripheral microtubule array to form the ciliary axoneme. The process of ciliogenesis is currently poorly understood due to the complexities of the underlying molecular machinery (Avasthi and Marshall, 2012; Ishikawa and Marshall, 2011). The ciliary proteome might comprise as many as 1,000 proteins (Boldt et al., 2016; Gherman et al., 2006; Mick et al., 2015) and many of these components likely play a role in the formation, extension, and regulation of cilia.

Cep104 is a highly relevant protein in this regard, and we attempted to elucidate its function by focusing on its domain architecture as an approach to understand its role in ciliogenesis. Cep104 has been shown to localize to the distal end of centrioles (Jiang et al., 2012; Satish Tammana et al., 2013) where it interacts through its N- and C-terminal region, respectively, with the distal end proteins Cep97 and CP110 (Jiang et al., 2012). Cep104 interacts with the microtubule end-binding protein EB1 through a C-terminal peptide motif (Jiang et al., 2012) and rides in vivo on the ends of elongating and shrinking ciliary axonemes (Jiang et al., 2012; Satish Tammana et al., 2013). Depletion of Cep104 leads either to the absence of cilia or the formation of cilia with a reduced length (Jiang et al., 2012; Satish Tammana et al., 2013). Furthermore, Cep104 mutations are linked to Joubert syndrome, a genetically heterogeneous ciliopathy (Srour et al., 2015). Taken together, these in vivo data suggest that Cep104 has an important role in regulating microtubule length in cilia. However, the molecular details of its activity are currently not known and it is unclear whether Cep104 exerts its function directly or indirectly through recruiting or regulating other ciliary factors.

Several kinases such as Aurora A, Plk1, and NIMA-related kinases such as Nek1 and Nek2 have been implicated in regulating the stability of cilia (Liang et al., 2016). Nek1 localizes to the basal body region (Shalom et al., 2008), and also to cilia when overexpressed as a kinase dead version (White and Quarmby, 2008). A role of Nek1 in cilia function is further suggested by the fact that mutations in Nek1 are linked to ciliopathies in vivo: polycystic kidney disease in mice (Upadhya et al., 2000) and short-rib thoracic dysplasia or oral-facial-digital syndrome type II in humans (El Hokayem et al., 2012; Monroe et al., 2016; Thiel et al., 2011). Mutations in Nek1 or its depletion lead to the formation of dysfunctional cilia that display structural aberrations such as branching (Shalom et al., 2008; Thiel et al., 2011; Wang et al., 2014), arguing that Nek1 might be involved in the stabilization of axonemal microtubules. In agreement with a role of Nek1 in cilia microtubule stabilization is that its protein levels decrease during the process of cilia resorption (Wang et al., 2014). The activity, expression levels, and localization of Nek1 probably have to be regulated tightly to enable efficient cilia formation, as both Nek1 overexpression (Shalom et al., 2008; White and Quarmby, 2008) and depletion (Wang et al., 2014) inhibit normal cilia formation.

Here we show that the Cep104 protein exhibits a complex, multi-domain organization. We have structurally characterized two of these domains, a central tubulin-binding TOG (tumor overexpressed gene) domain and a C-terminal domain consisting of an array of four Zn fingers (ZNF domain). Furthermore, we identify the kinase Nek1 as a novel binding partner of the C-terminal ZNF domain. We show that Nek1 competes for binding to this domain with the centriolar end-binding factor CP110.

 © 2016 MRC Laboratory of Molecular Biology. Published by Elsevier Ltd.
This is an open access article under the CC BY license (http://creativecommons.org/licenses/by/4.0/).

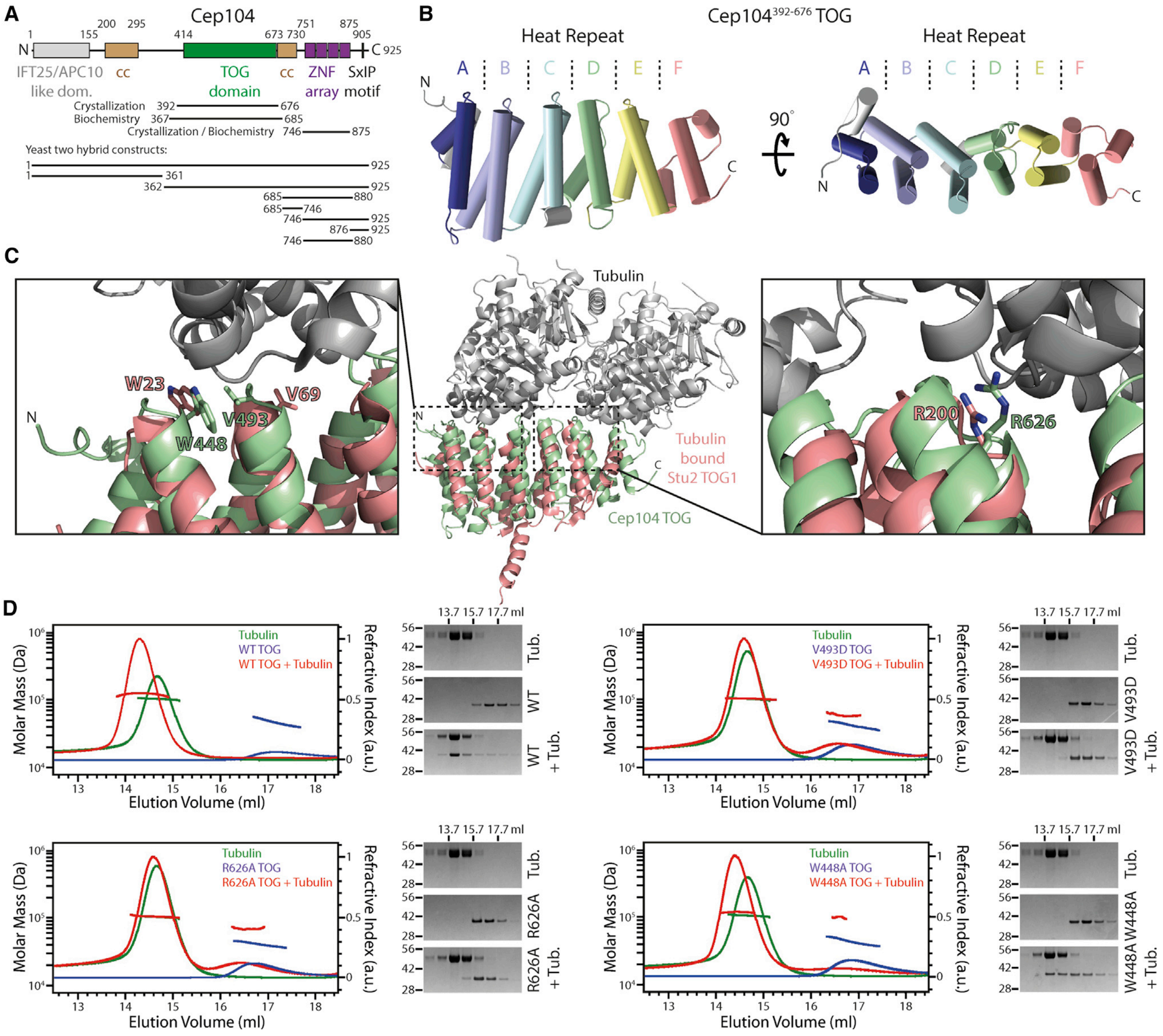

Figure 1. Cep104 Contains a Canonical Tubulin-Binding TOG Domain

(A) Domain overview of human Cep104. Lines indicate constructs that were used in this work.

(B) Ribbon representation of the Cep104 TOG structure. Helices are displayed as cylinders. The six HEAT repeats that constitute the TOG domain are colored individually. Note the partial distortion of HEAT repeat D and F and the slight curvature of the TOG domain.

(C) Middle: ribbon representation of TOG1 of yeast Stu2 (red) bound to a tubulin dimer (gray), PDB: 4ffb. Stu2 TOG1 is overlaid with our Cep104 TOG structure (green), showing the overall similarity of the TOG domain fold. Left and right: detailed views of the Stu2 TOG1-tubulin-binding interfaces with those residues labeled that were previously found to be critical for tubulin binding by Stu2. Note that these residues are conserved in Cep104 TOG and found in similar positions.

(D) Cep104 TOG binds tubulin in solution. SEC-MALS chromatograms of human Cep104 TOG constructs, run alone or in the presence of tubulin. The horizontal lines indicate the molar masses derived from the refractive index and light-scattering signals as described in Experimental Procedures. On the right side of the chromatograms, Coomassie-stained SDS-PAGE gels show the corresponding peak fractions. The approximate elution volumes are indicated above the gels. The tubulin-alone run is identical in all chromatograms and gels and is only shown repetitively to allow an easier comparison.

See also Figures S1 and S2.

Taken together, our data suggest that Cep104 regulates cilia length by facilitating tubulin addition to ciliary microtubules and might also be involved in regulating other factors, possibly by interacting with Nek1. Our findings reveal a novel link between ciliopathy-associated proteins and suggest a model of how Cep104 activity at microtubule ends is tied to ciliogenesis.

RESULTS

Cep104 Has a Complex Multi-Domain Organization

To identify regions in Cep104 that might be involved in its in vivo activities, we first analyzed the human Cep104 protein sequence using a set of bioinformatics methods (Figure 1A). Secondary

Table 1. SEC-MALS Analysis of Molecular Weights of Tubulin and Human Cep104 TOG Constructs, Run Alone or in Combination

Run	Derived Mw (kDa)	Theoretical Mw (kDa)	Polydispersity (Mw/Mn)
Tubulin alone	103	100	1.000
Tubulin + WT TOG (tubulin peak)	124	136 (1:1 complex)	1.000
WT TOG	46	36	1.010
Tubulin + R626A TOG (tubulin peak)	104	100 (tubulin alone)	1.000
R626A TOG	40	36	1.005
Tubulin + V493D TOG (tubulin peak)	103	100 (tubulin alone)	1.000
V493D TOG	41	36	1.008
Tubulin + W448A TOG (tubulin peak)	116	136 (1:1 complex)	1.000
W448A TOG	41	36	1.009

Mw, weight-average molecular weight; Mn, number-average molecular weight.

structure prediction using Jpred (Cole et al., 2008; Cuff and Barton, 2000) suggested the presence of an N-terminal all-β domain and a central all-α-helical domain. In addition, the multiple sequence alignment of Cep104 homologs revealed a number of invariant Cys and His residues in their C-terminal regions with a pattern reminiscent of classical C2H2 Zn fingers. These putative Zn fingers precede the previously identified SxIP motif that mediates binding to EB1 (Jiang et al., 2012). The N-terminal, central, and C-terminal domains are separated by two predicted coiled-coil regions as computed with COILS (Lupas et al., 1991). Each of these domains is well conserved in Metazoa with the exception of the SxIP motif that is absent in flies.

A PSI-BLAST search with the Cep104 N-terminal region, previously implicated in Cep97 binding (Jiang et al., 2012), retrieved hits to a number of structurally characterized domains of intraflagellar transport protein 25 (IFT25) (Bhogaraju et al., 2011), DNA repair protein XRCC1 (Cuneo and London, 2010), and anaphase-promoting complex APC subunit 10 (Au et al., 2002). These protein domains fold into a nine-stranded β sandwich with a jelly-roll topology and belong to a large superfamily, the founding member of which was a galactose-binding domain of fungal galactose oxidase. The homology to these domains was also confirmed with HHpred (Hildebrand et al., 2009; Soding, 2005), which gave hits to several members of this superfamily.

Our attempts to crystallize this region were unsuccessful. However, we have built a homology model using the structures of IFT25, APC10, and the N-terminal domain of XRCC1 as templates. Mapping of the sequence conservation onto our N-Cep104 model revealed a highly conserved region that could represent its binding site for Cep97 or other, undiscovered binding partners. Intriguingly, this region also colocates with the known protein-protein interaction sites of the structurally related IFT25 and APC10 and might therefore constitute a hot spot for protein-protein interactions in APC10-like domains (Figure S1).

A PSI-BLAST search with the central α-helical region gave matches to several TOG domains from CLASP (CLIP-associated protein) proteins, some of which with previously determined three-dimensional structures. TOG domains are typically implicated in tubulin binding, and we found residues critical for this binding to be conserved in Cep104 (Figures S2A and S2B). Thus, we decided to check whether the Cep104 TOG domain would also be capable of interacting with tubulin. To this end, we purified recombinant human Cep104 TOG and subjected it to size-exclusion chromatography coupled with multi-angle light scattering (SEC-MALS) in the presence or absence of tubulin. The results shown in Figure 1D and Table 1 demonstrate that Cep104 TOG is indeed a tubulin-binding domain and suggest a probable 1:1 complex between both proteins.

High-Resolution Structure of the Human Cep104 TOG Domain and Biochemical Characterization of Its Tubulin Binding

To gain a structural insight into the Cep104-tubulin interaction, we determined the high-resolution structure of the TOG domain to a resolution of 2.3 Å by X-ray crystallography (Figure 1B; Tables 2 and S1). Characteristic for TOG domains, the Cep104 TOG structure consists of six HEAT-like repeats A–F and globally superposes well with other known TOG structures (root-mean-square deviation [rmsd] 3–3.5 Å, Figure S2C). The individual repeats pack nearly parallel to each other creating an elongated domain with a slight left-handed curvature due to a left shift and twist of repeat D relative to repeat C. Canonical HEAT repeats consist of two antiparallel helices in which the polypeptide chain undergoes a near 90° bend following the C-terminal helix cap. Repeats A–C and E of the Cep104 TOG domain are canonical, whereas repeats D and F are partly distorted. A structure comparison of Cep104 TOG with the TOG domains from XMAP215 and CLASP proteins shows that the conformation of the loops that define their tubulin-binding interface is more conserved than those of the loops found on the opposite side. Furthermore, the residues on that side of the Cep104 TOG domain are highly conserved across different species (Figure S2D).

Structure superposition of the human Cep104 TOG domain with the Stu2 TOG1 domain in complex with tubulin suggest that key residues implicated in the Stu2-tubulin interaction (Ayaz et al., 2012) are conserved, such as the W448, V493, and R626 residues (Figure 1C). To test whether these residues are indeed crucial for tubulin binding, we introduced point mutations in Cep104 TOG, produced the corresponding recombinant proteins, and subjected them to SEC-MALS in the presence or absence of tubulin. The results shown in Figure 1D and Table 1 demonstrate that these mutants are indeed severely (V493D and R626A) or mildly (W448A) impaired in their ability to interact with tubulin. Thus, the Cep104 TOG domain engages tubulin in a similar way to the Stu2 TOG1 domain.

Identification of Nek1 as a Potential Binding Partner of the Cep104 ZNF Domain

Tubulin binding by Cep104 could explain its role in cilia elongation through regulating tubulin addition to microtubule ends, similar to the proposed mechanism for TOG domain containing microtubule-associated proteins (MAPs) (Akhmanova and Steinmetz, 2015). However, with a complex domain architecture, Cep104 might have additional roles at centriole and cilia ends.

Table 2. Dataset Analysis and Refinement Statistics

Construct	SeMet Human TOG Cep104$^{392-676}$ (Remote)	Human ZNF Cep104$^{746-875}$ S763E
Beamline	ID29 (ESRF)	MarDTB (in-house source)
Space group	P21	P212121
Wavelength (Å)	0.94	1.54
Monomers in the asymmetric unit	2	4
Unit cell dimensions (Å)	a = 52.3, b = 53.4, c = 155.4; α = 90.0, β = 91.9, γ = 90.0	a = 74.2, b = 80.3, c = 118.1; α = 90.0, β = 90.0, γ = 90.0
Resolution (Å)	49.5–2.3	30.31–1.8
Completeness (overall/inner/outer shell)	99.7/98.2/99.6	100.0/98.8/100.0
R_{merge} (overall/inner/outer shell)	0.130/0.041/1.066	0.075/0.031/1.395
R_{pim} (overall/inner/outer shell)	0.059/0.023/0.458	0.030/0.012/0.569
Mean I/σI (overall/inner/outer shell)	10.2/32.2/1.8	13.6/45.2/1.3
Multiplicity (overall/inner/outer shell)	6.9/6.5/7.1	7.1/6.8/7.0
Wilson B factor	33.5	33.1
No. of reflections (used in refinement)	40,229 (40,221)	66,109 (66,016)
No. of atoms	4,250	4,691
Waters	115	387
R_{work}/R_{free} (% data used)	23.4/25.3 (5.0%)	19.2/22.9 (4.8%)
Rmsd from ideal values: bond length/angles	0.008/1.282	0.007/0.809
Mean B value	51.4	45.2
Correlation coefficient F_o-F_c/F_o-F_c free	0.932/0.924	0.965/0.948
MolProbity score	0.85 (100th percentile)	1.08 (100th percentile)
MolProbity clashscore, all atoms	1.3	1.32
Poor rotamers (%)	0.2	0
Ramachandran outliers (%)	0	0
Ramachandran favored (%)	98.9	96.6
PDB entry code	5LPH	5LPI

See also Tables S1 and S2.

We therefore attempted to identify novel binding partners of Cep104. We first used BioID (Roux et al., 2012) to identify proteins in the vicinity of Cep104 in vivo. Thus, we expressed a BirA* (the R118G mutant of the biotin ligase BirA)-Cep104 fusion protein in tissue culture cells, purified the biotinylated proteins from cell lysates, and determined their identity by mass spectrometry. In agreement with a recent large-scale BioID profiling of components of the centrosome-cilium interface (Gupta et al., 2015), we found a number of centriolar and centrosomal proteins as proximity partners of Cep104 (Figures 2A and S3).

To establish which of these are candidates for a direct Cep104 interaction, we then used crosslinking of Cep104 in vivo and compared the two datasets. We tagged Cep104 N-terminally with wild-type BirA together with its site-specific biotinylation target sequence (AviTag) (Beckett et al., 1999) and expressed this construct in tissue culture cells. Subsequently, we prepared lysates from these cells, chemically crosslinked them, and isolated biotinylated Cep104 for mass-spectrometric analysis. To limit the background, we only considered those hits with a 10-fold enrichment of exclusive unique peptide counts compared with the control (Figure S3).

Under these conditions, only four hits were identified that were shared between the BioID and the crosslinking experiment, one of which was Nek1 kinase (Figure 2A). Nek1 locates to the basal body region and (when overexpressed as a kinase dead version) cilia (Shalom et al., 2008; White and Quarmby, 2008), is found in proximity to Cep104 (Gupta et al., 2015) and was recently also detected in Cep104 affinity-capture experiments in a high-throughput interactome study (Hein et al., 2015). Intriguingly, mutations in both Cep104 and Nek1 cause ciliopathies in humans: in the case of Cep104 Joubert syndrome (Srour et al., 2015) and in the case of Nek1 short-rib thoracic dystrophy or oral-facial-digital syndrome type II (El Hokayem et al., 2012; Monroe et al., 2016; Thiel et al., 2011). Both proteins play a role in cilia formation and/or length regulation (Jiang et al., 2012; Satish Tammana et al., 2013; Shalom et al., 2008; Thiel et al., 2011; Wang et al., 2014).

Nek1 and CP110 Binding to the Cep104 ZNF Domain Is Mutually Exclusive

To gain indications as to whether the Nek1-Cep104 interaction is direct, we first used a yeast two-hybrid assay. The results displayed in Figure 2B show that both proteins interacted within this assay. Further truncation experiments mapped this interaction to a region in the C-terminal half of Cep104 that comprises its array of Zn fingers. Supporting our yeast two-hybrid data, we also detected a ZNF-array-dependent Cep104-Nek1

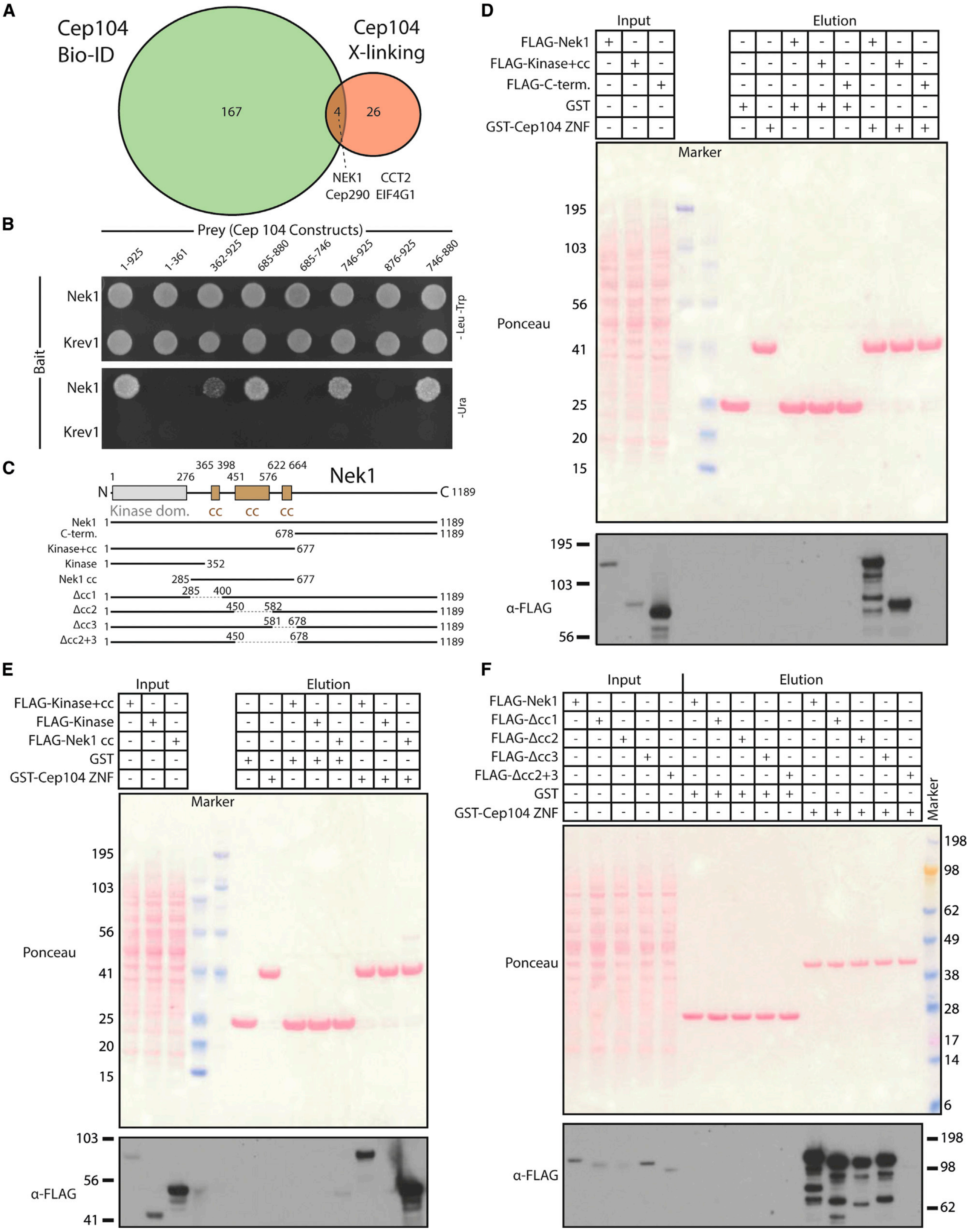

(legend on next page)

interaction in a rerouting and capture assay in tissue culture cells (Figure S4).

To fine-map this putative interaction further, we then used a pull-down assay with a glutathione S-transferase (GST)-tagged, recombinant version of the Cep104 ZNF array and different 3×FLAG-tagged Nek1 constructs that were transiently overexpressed in mammalian tissue culture cells. The results from our pull-down experiments shown in Figures 2C–2F confirm that Nek1 can interact with the Cep104 ZNF domain, and allowed us to define the coiled-coil but not the kinase domain of Nek1 as its main Cep104 ZNF-binding region. We found a corresponding part of Nek1 (residues 451–677) to also bind as a recombinant protein to the Cep104 ZNF array in vitro (Figure 3B).

Recently CP110 has been shown to interact with a section of Cep104 that contains its putative ZNF array, and the interacting region in CP110 has subsequently been mapped to $CP110^{907-936}$ (Jiang et al., 2012; Rezabkova et al., 2016). Given that both CP110 and Nek1 bind to the Cep104 ZNF array, we asked whether they share the same binding site or could engage their target sites on the Cep104 ZNF array simultaneously. To test this, we used a competition assay in which we assayed the ability of recombinant GST-Cep104 ZNF to pull down 3×FLAG-tagged Nek1 (transiently overexpressed in mammalian tissue culture cells) in the presence of increasing amounts of $CP110^{906-936}$. Our data shown in Figure 3A suggest that Nek1 and CP110 indeed compete for binding to the Cep104 ZNF array. Competition was specific to $CP110^{906-936}$, as it was not observed when we used a recombinantly produced peptide from the centriolar protein STIL ($STIL^{404-448}$, Figure S5).

To corroborate our findings, we also performed this assay with the recombinant Cep104-binding region of Nek1 (residues 451–677) and the binding peptide from CP110. We found that $Nek1^{451-677}$, but not $STIL^{404-448}$, competed with $CP110^{906-936}$ for binding to the Cep104 ZNF array (Figure 3B). Thus, we conclude that CP110 and Nek1 probably bind to either the same or spatially overlapping regions in Cep104.

High-Resolution Structure of the Zn Finger Tandem Repeats of Cep104

Our attempts to crystallize the Cep104 ZNF-Nek1 complex did not yield any crystals. However, we obtained crystals for the human apo-Cep104 ZNF domain (S763E mutant) and solved its structure by X-ray crystallography to a resolution of 1.8 Å (Figure 4; Tables 2 and S2). The S763E mutation was used since we found it to improve the solubility of the protein. The residue S763 is located at the loop connecting the β hairpin to the α helix of the first ZNF. This residue is exposed to the solvent and is poorly conserved (Figure 4A left, inset; Figure S6A). The Cep104 ZNF structure consists of four tandem C2HC Zn fingers that pack together to form a single globular domain that, to our knowledge, represents a novel, previously unobserved arrangement of Zn fingers.

The individual Zn fingers are variants of the classical C2H2 ZNFs in which the last Zn-coordinating residue is Cys instead of His. They consist of a β hairpin followed by an α helix that packs on it. The Zn ion is coordinated by two Cys residues located on the hairpin and by His and Cys residues from the helix with the exception of ZNF3 and ZNF4, in which the last Zn-coordinating Cys is located at a loop region downstream of the C-terminal helix cap (Figure 4A, left). The classical C2H2 Zn fingers and their variants are frequently found in tandem where the individual ZNFs can be separated by linkers of different length. A similar array of C2HC Zn fingers is seen in the structure of TRAF6 (PDB: 3HCS) (Yin et al., 2009) but, in contrast to the Cep104 ZNFs, the TRAF6 ZNFs are arranged in a linear fashion along the axis of the molecule with a degree of rotation between successive ZNFs that is probably determined by the fixed sequence separation between them (Figure S6B). In the Cep104 domain, the sequence separation between the individual ZNFs is variable and increases from two residues between ZNF1 and ZNF2 to eight residues between ZNF3 and ZNF4.

The Cys and His residues that coordinate the Zn are invariant across Cep104 homologs (Figure S6A). Apart from these, several hydrophobic residues are highly conserved and define the hydrophobic core of the domain by making extensive hydrophobic contacts with each other (Figure 4A, right; Figure S6A). These hydrophobic residues group into three hydrophobic clusters that together contribute to the integrity of the unit. The contacts in the first cluster are constituted by the side chains of Phe756, Tyr773, Leu780, and Val789 that are located in ZNF1 and ZNF2. In addition, Trp774 and the guanido group of Arg782 form a cation-π interaction. Residues Pro846, Leu847, Trp859, Leu863, Ile792, and Leu795 in ZNF2 and ZNF4 are part of the second hydrophobic cluster, whereas the third is formed by residues Leu799, Leu800, Phe809, and Ala818 from ZNF2 and ZNF3. The strict conservation of these residues suggests that the compact globular shape of this domain is not due to crystal packing but that the four C2HC Cep104 ZNFs probably function as an integral, single unit. Consistent with this notion is that this domain shows a continuous patch of surface conservation that probably constitutes a protein-protein interaction interface for its binding partners (Figure 4B). The in vacuo electrostatic surface potential of this patch reveals extensive charges, suggesting that charge interactions might contribute to its binding mode.

Figure 2. Nek1 Is a Potential Binding Partner of Cep104

(A) Summary of the (proximity) interactome of Cep104. Green circle, number of Cep104 proximity interactors obtained from mass-spectrometric identification of biotinylated proteins in Hek293 cells expressing BirA*-Cep104-GFP (BioID). Orange circle, number of hits from mass-spectrometric analysis of a pull-down experiment from Hek293 cell lysates expressing Avitag-BirA(WT)-Cep104-GFP, crosslinked with DTSSP. In both cases only hits with an enrichment of at least 10-fold compared with the corresponding negative control were considered. The overlap between the two circles shows the hits shared between both experiments.
(B) Nek1 interacts with Cep104 in a yeast two-hybrid assay. Yeast plates showing growth of yeast expressing the indicated Bait and Prey proteins. SC -Leu/-Trp plates select for the presence of Bait and Prey plasmid only, while SC -Ura plates select for positive yeast two-hybrid interactions.
(C) Domain overview of human Nek1, isoform 4. Lines indicate constructs that were used in the pull-down experiments probing the Nek1-Cep104 interaction shown in (D) to (F).
(D–F) The Nek1 interaction with Cep104 maps to its coiled-coil domain. Western blots showing pull-down experiments with GST or GST-Cep104 ZNF and lysates from Hek293 cells transiently overexpressing the 3×FLAG-tagged human Nek1 constructs indicated above the blot.
See also Figures S3 and S4.

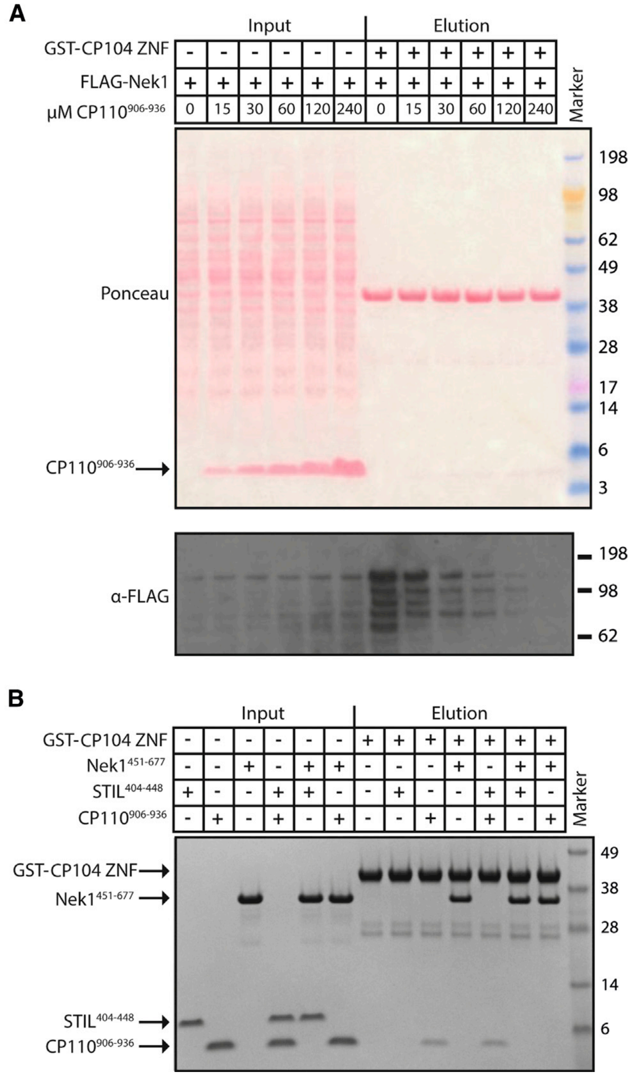

Figure 3. $CP110^{906–936}$ and Nek1 Binding to the Cep104 ZNF Array Is Mutually Exclusive

(A) Western blot showing a pull-down experiment with GST-Cep104 ZNF beads and lysates from Hek293 cells transiently overexpressing 3×FLAG-tagged human Nek1 in the presence of increasing concentrations of recombinant $CP110^{906-936}$.

(B) Coomassie-stained SDS-PAGE gel showing the results of an in vitro pull-down assay with recombinantly produced GST-Cep104-ZNF and $Nek1^{451-677}$ in the presence of $CP110^{906-936}$ or *Danio rerio* $STIL^{404-448}$.

See also Figure S5.

DISCUSSION

The process of ciliogenesis is currently poorly understood. Since Cep104 is a key protein involved in cilia formation and elongation, we subjected it to a bioinformatics, biochemical, and structural analysis. Our results show that human Cep104 contains a TOG domain that is structurally similar to other tubulin-binding TOG domains and engages tubulin through residues that are conserved between these. While our manuscript was in preparation, the TOG domain of chicken Cep104 was reported by another group (Rezabkova et al., 2016). Their TOG structure and analysis of the tubulin-interacting interface are in good agreement with our results.

Tubulin-binding TOG domains, e.g., in the XMAP215 family of MAPs, act to stabilize microtubules through facilitating tubulin addition to microtubule ends (Akhmanova and Steinmetz, 2015), and we speculate that the TOG domain in Cep104 has an analogous role. In support of this notion, Cep104 is found at the distal end of centrioles and cilia (Jiang et al., 2012; Satish Tammana et al., 2013) and its depletion results in absent or shortened cilia (Jiang et al., 2012; Satish Tammana et al., 2013). It is currently thought that the XMAP215 family of MAPs requires at least two TOG domains to function (Ayaz et al., 2014; Widlund et al., 2011), but our analysis revealed only a single TOG domain in Cep104. However, Cep104 contains two coiled-coil regions (Figure 1A), one of which forms dimers (Rezabkova et al., 2016). Thus, Cep104 might have two TOG domains that could function in vivo. However, tubulin binding by Cep104 could also have a different function during ciliogenesis. Cep104-bound tubulin could, for example, be transferred to other proteins or act as a signal during cilia formation.

Although inroads have been made, many questions concerning the regulation of cilia formation are still open. Intriguingly, we identified Nek1 as a novel interactor of Cep104. Several lines of evidence suggest that the Cep104-Nek1 interaction is physiologically relevant: Nek1 has been found in the proximity of Cep104 (Gupta et al., 2015 and our study), and was also detected in Cep104 affinity-capture experiments in a high-throughput interactome study (Hein et al., 2015). Furthermore, Nek1 and Cep104 are involved in the stabilization of the ciliary axoneme or in cilia formation and elongation (Jiang et al., 2012; Satish Tammana et al., 2013; Shalom et al., 2008; Thiel et al., 2011; Wang et al., 2014), and mutations in both cause ciliopathies (El Hokayem et al., 2012; Monroe et al., 2016; Srour et al., 2015; Thiel et al., 2011).

We found that Nek1 and CP110 compete for binding to the Cep104 ZNF domain through regions that are both basic (pI ~9.4/~10.4). Since our Cep104 ZNF structure revealed strikingly conserved and charged surface patches, electrostatic forces might contribute to their binding. However, in the absence of further structural information, the molecular details of the CP110/Nek1-Cep104 interaction are currently unclear.

The mutually exclusive binding of CP110 and Nek1 to Cep104 might play a role in cilia formation. Cep104 colocalizes with CP110 at the distal end of centrioles (Jiang et al., 2012; Satish Tammana et al., 2013). In the early stages of ciliogenesis CP110 becomes removed (Goetz et al., 2012; Spektor et al., 2007; Tanos et al., 2013), potentially liberating the Nek1-binding site on Cep104 and allowing Nek1 recruitment. The recruitment of critical kinases to centrioles/cilia has previously been described, e.g., the binding of TTBK2 kinase to the distal appendage protein Cep164 (Cajanek and Nigg, 2014; Oda et al., 2014). Cep104 might act analogously as a recruitment platform for Nek1 that could then, for example, promote substrate phosphorylation. Intriguingly, Nek1's Cep104 interacting region mapped to its predicted coiled-coil region that has previously been shown to interact with other proteins, such as the ciliary motor protein Kif3a (Surpili et al., 2003). Thus, binding competition could constitute a general feature of Nek1 regulation.

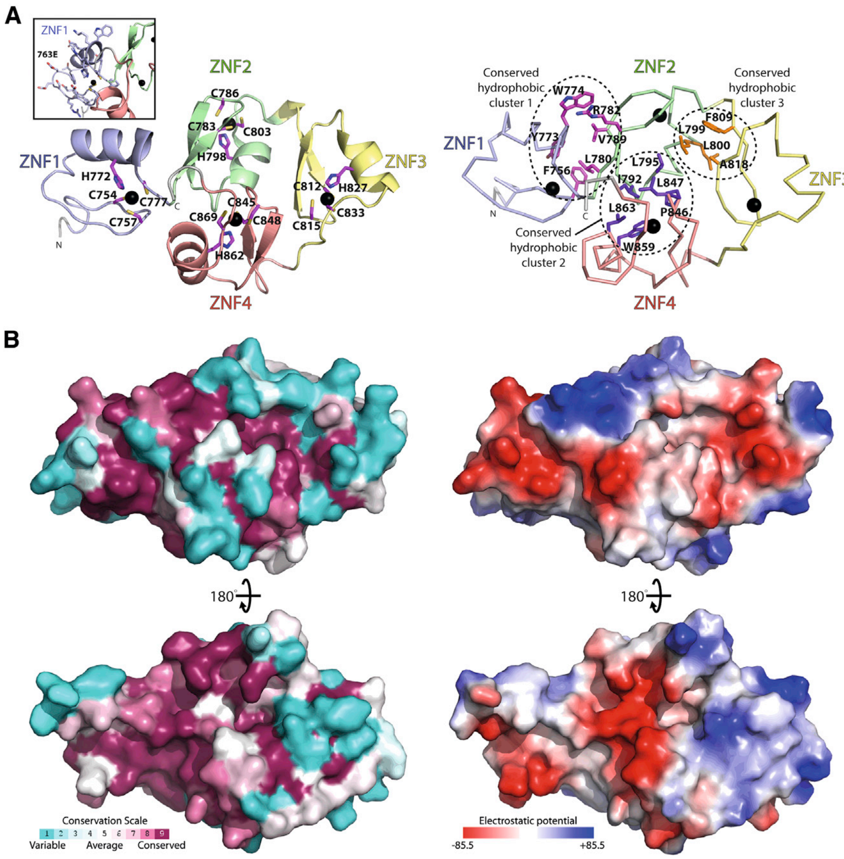

Figure 4. High-Resolution Structure of the Zn Finger Array of Cep104

(A) The Cep104 ZNFs adopt an overall globular domain. Left: ribbon presentation of the human Cep104 ZNF array structure (Cep104$^{746-875}$ S763E). The individual Zn fingers are colored distinctly. The Zn-coordinating side chains in the ZNFs are shown as sticks and labeled, the coordinated Zn ions are displayed as black spheres. Inset: close-up of ZNF1 with side chains displayed as sticks. Note that the S763E mutation that was used to improve solubility of the Cep104 ZNF domain is located in a loop and does not make contact with the rest of the domain. This residue is poorly conserved (Figure S6A). Right: similar view of the Cep104 ZNF domain. Labeled and shown as sticks are the side chains of the three conserved hydrophobic clusters in the interfaces between the individual ZNFs (Figure S6A). These clusters maintain the overall globular packing of the ZNF array.

(B) Top: similar view as in (A) but as a molecular surface colored according to CONSURF evolutionary conservation score (left) from unconserved (cyan) to highly conserved (burgundy), or colored according to in vacuo electrostatic potential (right) from positive (blue) to negative potential (red). Bottom: rotated 180° as indicated.

See also Figure S6.

Together with the established role of Cep104 in cilia length control and our description of a tubulin-interacting TOG domain in Cep104, we speculate that Cep104 might act to elongate cilia by facilitating tubulin addition. This activity of Cep104 might be aided by its recruitment of Nek1 that could positively or negatively influence other factors involved in this pathway. Further in vivo studies, guided by our structural and biochemical data, will be necessary to corroborate and distinguish between alternative models.

EXPERIMENTAL PROCEDURES

Clones

Cep104 constructs are based on the canonical human Cep104 sequence. Nek1 constructs and numbering are based on isoform 4 of human Nek1 (lacking residues 398–422 and 477–520 compared to the canonical Nek1 sequence).

Recombinant Protein Purification

DNA encoding human Cep104 TOG domain (Cep104$^{392-676}$: crystallization, Cep104$^{367-685}$: biochemistry) and Cep104$^{746-875}$ (ZNF) were cloned into a pET28-derived vector to create open reading frames with a PreScission protease-cleavable, N-terminal 6×His tag. Mutants were created by site-directed mutagenesis. Cep104$^{367-685}$ constructs were expressed in *Escherichia coli* Rosetta at 18°C. Cell lysates were prepared by sonication, centrifugally cleared, and subjected to Ni-nitrilotriacetic acid (NTA) (Qiagen) purification using standard methods. The Ni-NTA eluate was dialyzed in 10 mM PIPES (pH 6.9), 300 mM NaCl, and 1 mM DTT, the tag cut off with PreScission protease, the dialyzed protein diluted in buffer A (10 mM PIPES [pH 6.9], 20 mM NaCl, 1 mM DTT) and subsequently applied to a HiTrap SP column (GE Healthcare). The protein was eluted using a linear gradient from buffer A to buffer A and 1 M NaCl.

SeMet TOG Cep104$^{392-676}$ was expressed in supplemented M9 medium as described previously (van Breugel et al., 2014) and purified by Ni-NTA chromatography, PreScission protease cleavage, and size-exclusion chromatography in 10 mM PIPES (pH 6.9), 300 mM NaCl, and 1 mM DTT.

Cep104$^{746-875}$ S763E expression, cell lysis, and Ni-NTA purification were as described above. The Ni-NTA eluate was dialyzed in 10 mM Tris-HCl (pH 8.0), 50 mM NaCl, and 1 mM DTT, the tag cut off with PreScission protease, the protein applied to a HiTrap Q HP column (GE Healthcare), and eluted using a linear gradient to 10 mM Tris-HCl (pH 8.0), 1 mM DTT, and 1 M NaCl. Peak fractions were subjected to size-exclusion chromatography in 10 mM Tris-HCl (pH 8.0), 50 mM NaCl, and 1 mM DTT.

DNA encoding human Nek1$^{451-677}$ and CP110$^{906-936}$ were cloned into a modified pRSETa vector (Invitrogen) containing two His-tagged lipoyl domains (Cottee et al., 2013). Constructs were expressed in *E. coli* C41(DE3) and purified using Ni-NTA (Qiagen) chromatography using standard methods. The Ni-NTA eluates were dialyzed against 50 mM Tris-HCl (pH 8.0), 300 mM NaCl, and 5 mM imidazole (pH 7.5), the His-tagged lipoyl tags cut off with tobacco etch virus protease (Sigma), and removed by rebinding to Ni-NTA agarose (Qiagen). The flow-throughs were dialyzed and subjected to ion-exchange chromatography using linear NaCl gradients.

DNA encoding Cep104$^{746-875}$ (including an N-terminal linker, KKEGGSNQ TSLYKKAGSAAAPFTM) was cloned as a BamHI-EcoRI fragment into pGEX-6P1 (GE Healthcare). This construct or pGEX-6P1 alone were expressed in *E. coli* Rosetta at 18°C and purified using Glutathione Sepharose 4B (GE Healthcare). Eluates were applied to a HiTrap Q HP column (GE Healthcare) as described above for Cep104$^{746-875}$ S763E.

All proteins were concentrated, flash-frozen in liquid nitrogen, and stored at −80°C.

Cep104$^{392-676}$ TOG and Cep104$^{746-875}$ ZNF Crystallization

SeMet TOG Cep104$^{392-676}$ crystals were obtained by the vapor-diffusion method with a reservoir solution of 100 mM 2-(N-morpholino)ethanesulfonic acid (MES) (pH 6.0), 11% (w/v) polyethylene glycol (PEG)-20K at 19°C and using 100 nL of protein solution and 100 nL of reservoir solution. Crystals

were mounted after 2 days in 100 mM MES (pH 6.0) and 30% (v/v) PEG-400, and frozen in liquid nitrogen.

ZNF Cep104$^{746-875}$ S763E was crystallized at 4°C by the vapor-diffusion method with 100 nL of protein solution and 100 nL of the reservoir solution using the MORPHEUS screen (Gorrec, 2009) (MDL). Crystals were obtained in well A3 of that screen (reservoir solution: 10% [w/v] PEG-4000, 20% [v/v] glycerol, 0.1 M MES/imidazole [pH 6.5], 30 mM $MgCl_2$, 30 mM $CaCl_2$). Crystals were mounted after 15 days in cryoprotectant (reservoir solution to which 1/10 volume of 100% glycerol had been added) and were frozen in liquid nitrogen.

The protein concentrations of the crystallized constructs were determined by the Bradford assay with BSA as a standard, and were 25 mg/mL (SeMet TOG Cep104$^{392-676}$) and 24 mg/mL (ZNF Cep104$^{746-875}$ S763E).

Data Collection and Processing

Datasets were integrated using MOSFLM (Leslie and Powell, 2007) (ZNF Cep104$^{746-875}$ S763E) or XDS (Kabsch, 2010) (SeMet TOG Cep104$^{392-676}$). Datasets were scaled using SCALA (Evans, 2006). The TOG Cep104$^{392-676}$ structure was solved by MAD from a 3-wavelength SeMet dataset using the SHELX CDE pipeline (Sheldrick, 2008). BUCCANEER (Cowtan, 2006) and manual building were employed to build an initial model. REFMAC (Murshudov et al., 2011) was used to refine the model against the remote dataset with manual building done in Coot (Emsley and Cowtan, 2004). The ZNF Cep104$^{746-875}$ S763E structure was solved by Zn MAD from a synchrotron (I04, Diamond Light Source)-collected dataset using the SHELX CDE pipeline (Sheldrick, 2008), resulting in a main-chain autotraced model. This model was subsequently used as a search model for molecular replacement in PHASER (McCoy et al., 2007) with a higher-resolution in-house collected dataset. After autobuilding with BUCCANEER (Cowtan, 2006), the structure was refined in PHENIX.REFINE (Afonine et al., 2012), with manual building done in Coot (Emsley and Cowtan, 2004).

Cep104 BioID and Crosslinking

Constructs containing BirA*(R118G)-Cep104-eGFP (for BioID) or Avitag-WT BirA-Cep104-eGFP (for crosslinking) were cloned into pcDNA5 FRT/TO (Thermo Fisher Scientific) and integrated into Hek293 Trex Flpin cells by site-specific recombination using cotransfection with plasmid pOG44 (Thermo Fisher Scientific). Positive transformants were selected for with DMEM, 10% tetracycline-free fetal bovine serum, 200 μg/mL hygromycin, and 15 μg/mL blasticidin. For BioID two 150-cm^2 dishes at ~70% confluency were induced with 3 μg/mL tetracycline in selection medium containing 200 μM biotin for 24 hr, processed as previously described (Roux et al., 2012), and subjected to SDS-PAGE and mass-spectrometric analysis. As a control for the BioID experiment we used Hek293 Trex Flpin cells with an integrated construct that was not tagged with BirA.

In a similar setup, for the crosslinking experiment eight tetracycline-induced, biotin-supplemented 150-cm^2 dishes at ~70% confluency were used. Cells were washed with PBS, then lysed by sonication in 40 mL of 50 mM Na-phosphate (pH 7.5), 100 mM NaCl, one pill of Complete Protease Inhibitor (EDTA free, Roche), and 50 mM Na-phosphate (pH 7.5), 100 mM NaCl added to 75 mL. To 37.5 mL of this lysate was added 50 mg of 3,3′-dithiobis(sulfosuccinimidyl propionate) (DTSSP; Cambridge Bioscience) followed by incubation for 2 hr at 4°C. Subsequently, NaCl, Tris-HCl (pH 7.4), SDS, and EDTA were added to 0.5 M, 50 mM, 0.4%, and 5 mM, respectively. After a short incubation, Triton X-100 was added to 2% followed by one volume of 50 mM Tris-HCl (pH 7.4). After centrifugation (4.6 krpm, 30 min, 4°C) the supernatants were bound to streptavidin beads and the beads processed as described for BioID (Roux et al., 2012). Crosslinks were reversed by addition of 10 μL of 1 M DTT to the beads and incubation for 10 min at 50°C, before addition of biotin-saturated Laemmli buffer. Eluates were subjected to SDS-PAGE and mass-spectrometric analysis.

GST-Cep104 ZNF Domain, 3×FLAG-Nek1 Pull-Down Assay

Thirty micrograms of purified GST or GST-Cep104$^{746-875}$ were bound to Glutathione Sepharose 4B (GE Healthcare) beads in lysis buffer (50 mM Na-phosphate [pH 7.5], 100 mM NaCl, 1 mM DTT, and 0.1% [v/v] Nonidet P-40, supplemented with Complete Protease Inhibitor [EDTA free, Roche]). Beads were subsequently washed with lysis buffer and incubated at 4°C for 1 hr with 1 mL of centrifugally cleared cell lysates (prepared by sonication in lysis buffer) from Hek293 Trex Flpin cells transfected for ~2 days by Fugene6 (Promega) with 3×FLAG human Nek1 constructs (cloned into a pcDNA3.1 derivative, a kind gift of Manu Hegde). Beads were washed with lysis buffer, eluted with lysis buffer and 100 mM glutathione (pH 7.5), and the eluates subjected to SDS-PAGE and western blotting using anti-FLAG M2 mouse monoclonal antibody (Sigma).

For the competition assay with CP110$^{906-936}$, 109 μL of centrifugally cleared cell lysates from Hek293 Trex Flpin cells transfected with 3×FLAG-tagged human Nek1 were mixed with 16 μL of PBS, 1 mM DTT, and 0.1% IGEPAL CA-630 (octylphenoxypolyethoxyethanol) containing increasing amounts of CP110$^{906-936}$. After centrifugal clearing, the supernatants were added to GST-Cep104$^{746-875}$ beads and the binding experiments continued as described above.

In Vitro Competition Assay

Thirty micrograms of purified GST-Cep104$^{746-875}$ (ZNF) was bound to Glutathione Sepharose 4B (GE Healthcare) beads in binding buffer (PBS, 0.1% IGEPAL CA-630, 1 mM DTT). Beads were washed and incubated at 4°C for 1 hr with 100 μL of Nek1$^{451-677}$ (26 μM), CP110$^{906-936}$ (66 μM), *Danio rerio* STIL$^{404-448}$ (127 μM), or their binary combinations in binding buffer. Beads were washed three times and eluted with Laemmli buffer, and the eluates subsequently subjected to SDS-PAGE and Coomassie staining.

Yeast Two-Hybrid Assay

Constructs were cloned into vector pENTR/D-TOPO and subsequently transferred into vectors pDEST32 (Bait) or pDEST22 (Prey) using Gateway LR Clonase II (Thermo Fisher Scientific). Combinations of Bait and Prey plasmids were cotransformed into yeast strain MaV203 (Thermo Fisher Scientific) using standard methods and plated onto SC -Leu -Trp plates. Subsequently, colonies were inoculated into SC -Leu -Trp medium and grown overnight at 30°C before diluting and spotting them onto SC -Leu -Trp and SC -Ura plates. Plates were incubated for 3 days at 30°C.

Size-Exclusion Chromatography Coupled with Multi-Angle Light Scattering

WT and mutant TOG proteins were mixed with reconstituted tubulin (Cytoskeleton) in SEC running buffer (25 mM Tris-HCl [pH 7.5], 100 mM NaCl, 1 mM $MgCl_2$, 1 mM EGTA) to a final concentration of ~42 μM in a 1:1 stoichiometry. Protein samples were subsequently resolved and analyzed by SEC-MALS (in SEC running buffer) as described previously (van Breugel et al., 2014).

Bioinformatics

NCBI-NR was searched using PSI-BLAST (Altschul et al., 1997) to identify sequence homologs of Cep104. Selected sequences were aligned, after which the alignment was manually corrected and used as input for calculation of conservation scores. Multiple sequence alignments were produced with MAFFT (Katoh and Standley, 2013), and visualized using JALVIEW (Waterhouse et al., 2009). Secondary structure predictions were computed with Jpred (Cole et al., 2008; Cuff and Barton, 2000) and coiled coils were predicted with COILS (Lupas et al., 1991). Evolutionary conservation was computed using CONSURF (Ashkenazy et al., 2010). HHPRED (Hildebrand et al., 2009) was used to search the PDB and Structural Classification of Proteins databases for structural homologs. Homology models were generated with MODELLER (Sali and Blundell, 1993) using the structures 1xna, 1xnt, 1gqp, 1tvg, 2yc4, and 3k75 as templates and manually optimized alignments as inputs. Structure-based multiple sequence alignments were produced manually using pairwise superpositions computed with TOPMATCH (Sippl and Wiederstein, 2008).

Mass Spectrometry

The protein-containing polyacrylamide gel slices (1–2 mm) were prepared and subjected to mass-spectrometric analysis as described previously (Staples et al., 2014).

SUPPLEMENTAL INFORMATION

Supplemental Information includes six figures and two tables and can be found with this article online at http://dx.doi.org/10.1016/j.str.2016.11.014.

AUTHOR CONTRIBUTIONS

C.Al-J., M.Y., and M.v.B. crystallized the constructs and/or collected the data and solved their X-ray structures. M.v.B. performed the CP110-Nek1 competition assays, the pull-down experiments, and the BioID/crosslinking experiments. D.D.B. carried out the yeast two-hybrid and the "rerouting and capture" assays, and analyzed the BioID/crosslinking data. S.H.M. and C.M.J. performed/analyzed the SEC-MALS experiments. A.A. did the bioinformatics and structural analyses. Proteins were purified by C.Al-J. and M.v.B. All authors contributed to the writing of the manuscript.

ACKNOWLEDGMENTS

For beamline support, we would like to acknowledge Dr. McCarthy (ID29) at the European Synchrotron Radiation Facility (ESRF), Grenoble, France and Carina Lobley (I04) at the Diamond Light Source, Didcot, UK. We are grateful to Farida Begum and Mark Skehel (mass spectrometry facility, MRC-LMB, Cambridge, UK) for their mass-spectrometric analyses. We thank Manu Hegde, MRC-LMB, Cambridge, UK for the kind gift of Hek293 Trex Flpin cells as well as for the gift of a pcDNA3.1 derivative plasmid allowing the expression of N-terminally 3×FLAG-tagged constructs in mammalian cells. This work was supported by the Medical Research Council (MRC file reference MC_UP_1201/3).

Received: August 14, 2016
Revised: November 4, 2016
Accepted: November 18, 2016
Published: December 22, 2016

REFERENCES

Afonine, P.V., Grosse-Kunstleve, R.W., Echols, N., Headd, J.J., Moriarty, N.W., Mustyakimov, M., Terwilliger, T.C., Urzhumtsev, A., Zwart, P.H., and Adams, P.D. (2012). Towards automated crystallographic structure refinement with phenix.refine. Acta Crystallogr. D Biol. Crystallogr. *68*, 352–367.

Akhmanova, A., and Steinmetz, M.O. (2015). Control of microtubule organization and dynamics: two ends in the limelight. Nat. Rev. Mol. Cell Biol. *16*, 711–726.

Altschul, S.F., Madden, T.L., Schaffer, A.A., Zhang, J., Zhang, Z., Miller, W., and Lipman, D.J. (1997). Gapped BLAST and PSI-BLAST: a new generation of protein database search programs. Nucleic Acids Res. *25*, 3389–3402.

Ashkenazy, H., Erez, E., Martz, E., Pupko, T., and Ben-Tal, N. (2010). ConSurf 2010: calculating evolutionary conservation in sequence and structure of proteins and nucleic acids. Nucleic Acids Res. *38*, W529–W533.

Au, S.W., Leng, X., Harper, J.W., and Barford, D. (2002). Implications for the ubiquitination reaction of the anaphase-promoting complex from the crystal structure of the Doc1/Apc10 subunit. J. Mol. Biol. *316*, 955–968.

Avasthi, P., and Marshall, W.F. (2012). Stages of ciliogenesis and regulation of ciliary length. Differentiation *83*, S30–S42.

Ayaz, P., Ye, X., Huddleston, P., Brautigam, C.A., and Rice, L.M. (2012). A TOG:alphabeta-tubulin complex structure reveals conformation-based mechanisms for a microtubule polymerase. Science *337*, 857–860.

Ayaz, P., Munyoki, S., Geyer, E.A., Piedra, F.A., Vu, E.S., Bromberg, R., Otwinowski, Z., Grishin, N.V., Brautigam, C.A., and Rice, L.M. (2014). A tethered delivery mechanism explains the catalytic action of a microtubule polymerase. Elife *3*, e03069.

Beckett, D., Kovaleva, E., and Schatz, P.J. (1999). A minimal peptide substrate in biotin holoenzyme synthetase-catalyzed biotinylation. Protein Sci. *8*, 921–929.

Bhogaraju, S., Taschner, M., Morawetz, M., Basquin, C., and Lorentzen, E. (2011). Crystal structure of the intraflagellar transport complex 25/27. EMBO J. *30*, 1907–1918.

Boldt, K., van Reeuwijk, J., Lu, Q., Koutroumpas, K., Nguyen, T.M., Texier, Y., van Beersum, S.E., Horn, N., Willer, J.R., Mans, D.A., et al. (2016). An organelle-specific protein landscape identifies novel diseases and molecular mechanisms. Nat. Commun. *7*, 11491.

Cajanek, L., and Nigg, E.A. (2014). Cep164 triggers ciliogenesis by recruiting Tau tubulin kinase 2 to the mother centriole. Proc. Natl. Acad. Sci. USA *111*, E2841–E2850.

Cole, C., Barber, J.D., and Barton, G.J. (2008). The Jpred 3 secondary structure prediction server. Nucleic Acids Res. *36*, W197–W201.

Cottee, M.A., Muschalik, N., Wong, Y.L., Johnson, C.M., Johnson, S., Andreeva, A., Oegema, K., Lea, S.M., Raff, J.W., and van Breugel, M. (2013). Crystal structures of the CPAP/STIL complex reveal its role in centriole assembly and human microcephaly. Elife *2*, e01071.

Cowtan, K. (2006). The Buccaneer software for automated model building. 1. Tracing protein chains. Acta Crystallogr. D Biol. Crystallogr. *62*, 1002–1011.

Cuff, J.A., and Barton, G.J. (2000). Application of multiple sequence alignment profiles to improve protein secondary structure prediction. Proteins *40*, 502–511.

Cuneo, M.J., and London, R.E. (2010). Oxidation state of the XRCC1 N-terminal domain regulates DNA polymerase beta binding affinity. Proc. Natl. Acad. Sci. USA *107*, 6805–6810.

El Hokayem, J., Huber, C., Couve, A., Aziza, J., Baujat, G., Bouvier, R., Cavalcanti, D.P., Collins, F.A., Cordier, M.P., Delezoide, A.L., et al. (2012). NEK1 and DYNC2H1 are both involved in short rib polydactyly Majewski type but not in Beemer Langer cases. J. Med. Genet. *49*, 227–233.

Emsley, P., and Cowtan, K. (2004). Coot: model-building tools for molecular graphics. Acta Crystallogr. D Biol. Crystallogr. *60*, 2126–2132.

Evans, P. (2006). Scaling and assessment of data quality. Acta Crystallogr. D Biol. Crystallogr. *62*, 72–82.

Gherman, A., Davis, E.E., and Katsanis, N. (2006). The ciliary proteome database: an integrated community resource for the genetic and functional dissection of cilia. Nat. Genet. *38*, 961–962.

Goetz, S.C., Liem, K.F., Jr., and Anderson, K.V. (2012). The spinocerebellar ataxia-associated gene Tau tubulin kinase 2 controls the initiation of ciliogenesis. Cell *151*, 847–858.

Gorrec, F. (2009). The MORPHEUS protein crystallization screen. J. Appl. Crystallogr. *42*, 1035–1042.

Gupta, G.D., Coyaud, E., Goncalves, J., Mojarad, B.A., Liu, Y., Wu, Q., Gheiratmand, L., Comartin, D., Tkach, J.M., Cheung, S.W., et al. (2015). A dynamic protein interaction landscape of the human centrosome-cilium interface. Cell *163*, 1484–1499.

Hein, M.Y., Hubner, N.C., Poser, I., Cox, J., Nagaraj, N., Toyoda, Y., Gak, I.A., Weisswange, I., Mansfeld, J., Buchholz, F., et al. (2015). A human interactome in three quantitative dimensions organized by stoichiometries and abundances. Cell *163*, 712–723.

Hildebrand, A., Remmert, M., Biegert, A., and Soding, J. (2009). Fast and accurate automatic structure prediction with HHpred. Proteins *77* (*Suppl 9*), 128–132.

Ishikawa, H., and Marshall, W.F. (2011). Ciliogenesis: building the cell's antenna. Nat. Rev. Mol. Cell Biol. *12*, 222–234.

Jiang, K., Toedt, G., Montenegro Gouveia, S., Davey, N.E., Hua, S., van der Vaart, B., Grigoriev, I., Larsen, J., Pedersen, L.B., Bezstarosti, K., et al. (2012). A Proteome-wide screen for mammalian SxIP motif-containing microtubule plus-end tracking proteins. Curr. Biol. *22*, 1800–1807.

Kabsch, W. (2010). XDS. Acta Crystallogr. D Biol. Crystallogr. *66*, 125–132.

Katoh, K., and Standley, D.M. (2013). MAFFT multiple sequence alignment software version 7: improvements in performance and usability. Mol. Biol. Evol. *30*, 772–780.

Leslie, A.G.W., and Powell, H.R. (2007). Processing diffraction data with Mosflm. In Evolving Methods for Macromolecular Crystallography, *245* (Springer), pp. 41–51.

Liang, Y., Meng, D., Zhu, B., and Pan, J. (2016). Mechanism of ciliary disassembly. Cell Mol. Life Sci. *73*, 1787–1802.

Lupas, A., Van Dyke, M., and Stock, J. (1991). Predicting coiled coils from protein sequences. Science *252*, 1162–1164.

McCoy, A.J., Grosse-Kunstleve, R.W., Adams, P.D., Winn, M.D., Storoni, L.C., and Read, R.J. (2007). Phaser crystallographic software. J. Appl. Crystallogr. *40*, 658–674.

Mick, D.U., Rodrigues, R.B., Leib, R.D., Adams, C.M., Chien, A.S., Gygi, S.P., and Nachury, M.V. (2015). Proteomics of primary cilia by proximity labeling. Dev. Cell *35*, 497–512.

Monroe, G.R., Kappen, I.F., Stokman, M.F., Terhal, P.A., van den Boogaard, M.H., Savelberg, S.M., van der Veken, L.T., van Es, R.J., Lens, S.M., Hengeveld, R.C., et al. (2016). Compound heterozygous NEK1 variants in two siblings with oral-facial-digital syndrome type II (Mohr syndrome). Eur. J. Hum. Genet. *24*, 1752–1760.

Murshudov, G.N., Skubak, P., Lebedev, A.A., Pannu, N.S., Steiner, R.A., Nicholls, R.A., Winn, M.D., Long, F., and Vagin, A.A. (2011). REFMAC5 for the refinement of macromolecular crystal structures. Acta Crystallogr. D Biol. Crystallogr. *67*, 355–367.

Oda, T., Chiba, S., Nagai, T., and Mizuno, K. (2014). Binding to Cep164, but not EB1, is essential for centriolar localization of TTBK2 and its function in ciliogenesis. Genes Cells *19*, 927–940.

Rezabkova, L., Kraatz, S.H., Akhmanova, A., Steinmetz, M.O., and Kammerer, R.A. (2016). Biophysical and structural characterization of the centriolar protein Cep104 interaction network. J. Biol. Chem. *291*, 18496–18504.

Roux, K.J., Kim, D.I., Raida, M., and Burke, B. (2012). A promiscuous biotin ligase fusion protein identifies proximal and interacting proteins in mammalian cells. J. Cell Biol. *196*, 801–810.

Sali, A., and Blundell, T.L. (1993). Comparative protein modelling by satisfaction of spatial restraints. J. Mol. Biol. *234*, 779–815.

Satish Tammana, T.V., Tammana, D., Diener, D.R., and Rosenbaum, J. (2013). Centrosomal protein CEP104 (Chlamydomonas FAP256) moves to the ciliary tip during ciliary assembly. J. Cell Sci. *126*, 5018–5029.

Shalom, O., Shalva, N., Altschuler, Y., and Motro, B. (2008). The mammalian Nek1 kinase is involved in primary cilium formation. FEBS Lett. *582*, 1465–1470.

Sheldrick, G.M. (2008). A short history of SHELX. Acta Crystallogr. A. *64*, 112–122.

Sippl, M.J., and Wiederstein, M. (2008). A note on difficult structure alignment problems. Bioinformatics *24*, 426–427.

Soding, J. (2005). Protein homology detection by HMM-HMM comparison. Bioinformatics *21*, 951–960.

Spektor, A., Tsang, W.Y., Khoo, D., and Dynlacht, B.D. (2007). Cep97 and CP110 suppress a cilia assembly program. Cell *130*, 678–690.

Srour, M., Hamdan, F.F., McKnight, D., Davis, E., Mandel, H., Schwartzentruber, J., Martin, B., Patry, L., Nassif, C., Dionne-Laporte, A., et al. (2015). Joubert syndrome in French Canadians and identification of mutations in CEP104. Am. J. Hum. Genet. *97*, 744–753.

Staples, C.J., Myers, K.N., Beveridge, R.D., Patil, A.A., Howard, A.E., Barone, G., Lee, A.J., Swanton, C., Howell, M., Maslen, S., et al. (2014). Ccdc13 is a novel human centriolar satellite protein required for ciliogenesis and genome stability. J. Cell Sci. *127*, 2910–2919.

Surpili, M.J., Delben, T.M., and Kobarg, J. (2003). Identification of proteins that interact with the central coiled-coil region of the human protein kinase NEK1. Biochemistry *42*, 15369–15376.

Tanos, B.E., Yang, H.J., Soni, R., Wang, W.J., Macaluso, F.P., Asara, J.M., and Tsou, M.F. (2013). Centriole distal appendages promote membrane docking, leading to cilia initiation. Genes Dev. *27*, 163–168.

Thiel, C., Kessler, K., Giessl, A., Dimmler, A., Shalev, S.A., von der Haar, S., Zenker, M., Zahnleiter, D., Stoss, H., Beinder, E., et al. (2011). NEK1 mutations cause short-rib polydactyly syndrome type Majewski. Am. J. Hum. Genet. *88*, 106–114.

Upadhya, P., Birkenmeier, E.H., Birkenmeier, C.S., and Barker, J.E. (2000). Mutations in a NIMA-related kinase gene, Nek1, cause pleiotropic effects including a progressive polycystic kidney disease in mice. Proc. Natl. Acad. Sci. USA *97*, 217–221.

van Breugel, M., Wilcken, R., McLaughlin, S.H., Rutherford, T.J., and Johnson, C.M. (2014). Structure of the SAS-6 cartwheel hub from *Leishmania major*. Elife *3*, e01812.

Wang, W., Wu, T., and Kirschner, M.W. (2014). The master cell cycle regulator APC-Cdc20 regulates ciliary length and disassembly of the primary cilium. Elife *3*, e03083.

Waterhouse, A.M., Procter, J.B., Martin, D.M., Clamp, M., and Barton, G.J. (2009). Jalview Version 2—a multiple sequence alignment editor and analysis workbench. Bioinformatics *25*, 1189–1191.

White, M.C., and Quarmby, L.M. (2008). The NIMA-family kinase, Nek1 affects the stability of centrosomes and ciliogenesis. BMC Cell Biol. *9*, 29.

Widlund, P.O., Stear, J.H., Pozniakovsky, A., Zanic, M., Reber, S., Brouhard, G.J., Hyman, A.A., and Howard, J. (2011). XMAP215 polymerase activity is built by combining multiple tubulin-binding TOG domains and a basic lattice-binding region. Proc. Natl. Acad. Sci. USA *108*, 2741–2746.

Yin, Q., Lin, S.C., Lamothe, B., Lu, M., Lo, Y.C., Hura, G., Zheng, L., Rich, R.L., Campos, A.D., Myszka, D.G., et al. (2009). E2 interaction and dimerization in the crystal structure of TRAF6. Nat. Struct. Mol. Biol. *16*, 658–666.

Structure, Volume *25*

Supplemental Information

The Ciliopathy-Associated Cep104 Protein Interacts with Tubulin and Nek1 Kinase

Caezar Al-Jassar, Antonina Andreeva, Deepak D. Barnabas, Stephen H. McLaughlin, Christopher M. Johnson, Minmin Yu, and Mark van Breugel

Table S1, related to Table 2

Human TOG Cep104$^{392\text{-}676}$ SeMet MAD dataset analysis.

Beamline	ID29 (ESRF)	
Space Group	P21	
Wavelength (Å)	0.97916 (peak)	0.97923 (inflection)
Unit Cell dimensions (Å)	a=51.8 b=53.3 c=154.4 α=90.0 β=91.8 γ=90.0	a=52.1 b=53.3 c=155.4 α=90.0 β=92.0 γ=90.0
Resolution (Å)	49.5 – 2.3	49.54 – 2.3
Completeness (overall / inner / outer shell)	99.6 / 99.1 / 99.5	99.5 / 98.7 / 99.5
Rmerge (overall / inner / outer shell)	0.145 / 0.045 / 1.050	0.127 / 0.034 / 0.987
Rpim (overall / inner / outer shell)	0.069 / 0.031 / 0.456	0.060 / 0.025 / 0.426
Mean I/σI (overall / inner / outer shell)	9.2 / 30.4 / 1.8	10.2 / 32.2 / 2.1
Multiplicity (overall / inner / outer shell)	6.9 / 6.5 / 7.1	6.9 / 6.5 / 7.1
Se sites found / expected	15 / 22 (16 SeMet residues are visible in the electron density map)	

Table S2, related to Table 2

Human ZNF Cep104$^{746\text{-}875}$ S763E Zn MAD dataset analysis.

Beamline	I04 (Diamond Light Source)		
Space Group	P212121		
Wavelength (Å)	1.28295 (peak)	1.28321 (inflection)	1.21984 (remote)
Unit Cell dimensions (Å)	a=73.9 b=80.5 c=118.3 α=90.0 β=90.0 γ=90.0	a=73.9 b=80.5 c=118.3 α=90.0 β=90.0 γ=90.0	a=73.9 b=80.5 c=118.3 α=90.0 β=90.0 γ=90.0
Resolution (Å)	80.5 – 2.2	80.5 – 2.2	80.5 – 2.2
Completeness (overall / inner / outer shell)	99.2 / 99.7 / 98.1	99.3 / 99.8 / 98.2	99.4 / 99.5 / 98.5
Rmerge (overall / inner / outer shell)	0.139 / 0.069 / 1.512	0.114 / 0.067 / 1.017	0.198 / 0.067 / 1.976
Rpim (overall / inner / outer shell)	0.046 / 0.024 / 0.510	0.038 / 0.025 / 0.342	0.065 / 0.024 / 0.657
Mean I/σI (overall / inner / outer shell)	11.3 / 30.5 / 1.9	12.0 / 28.3 / 2.5	9.6 / 28.4 / 1.8
Multiplicity (overall / inner / outer shell)	9.9 / 8.5 / 9.4	10.1 / 8.7 / 9.6	10.1 / 8.7 / 9.8
Zn found / expected	6 / 16		

Figure S1, related to Figure 1A

Homology model of the N-terminal APC10-like domain of Cep104.

A) Ribbon presentation of the N-terminal domain of Cep104 (rainbow-coloured from N- to C-terminus) together with an equivalent representation as molecular surface, coloured according to Consurf evolutionary conservation score from unconserved (cyan) to highly conserved (burgundy). **B)** Equivalent view onto the IFT25-IFT27 (pdb 2yc2) and the APC10-HSL1 (pdb 5g04) complexes. Note that IFT27 and HSL1 engage their corresponding APC10(-like) domain at a region that is conserved in the N-terminal, APC10-like domain in Cep104.

Figure S2, related to Figure 1B-D

Multiple sequence alignments and structural analyses of Cep104's TOG domain.

A) A structure-based, multiple sequence alignment of Cep104 TOG and other TOG domains with experimentally determined high-resolution structures (pdb 4ffb Stu2 TOG1, pdb 4g3a Mast/Orbit TOG1, pdb 2qk1 Stu2 TOG2, pdb 2qk2 Msps TOG2, pdb 4qmj ch-TOG TOG4, pdb 2of3 Zyg9 TOG3, pdb 3woy CLASP2 TOG2. Residues that were mutated in this study are marked with green circles. Structurally equivalent residues are shown in upper case. **B)** Multiple sequence alignment of Cep104 TOG of closely related homologs. Residues that were mutated in this study are marked with green circles. **C)** The tubulin-binding interface of Cep104 TOG is conserved and conformationally similar to other tubulin binding TOG domains. Globally fitted overlay of the ribbon representation of the Cep104 TOG structure with other TOG domains with experimentally determined high-resolution structures. Helices are displayed as cylinders. The top view onto the tubulin-binding interface reveals similar positions of the α-helices and their connecting loops, while that is not observed when the domain is viewed from the bottom. Used PDB codes were: 4ffb (Stu2 TOG1), 2of3 (Zyg9 TOG3), 2qk1 (Stu2 TOG2), 2qk2 (Msps TOG2), 3woy (CLASP2 TOG2), 4g3a (Mast/Orbit TOG1), 4qmj (ch-TOG TOG4). **D)** Cep104 TOG structure displayed as molecular surface coloured according to Consurf evolutionary conservation score from unconserved (cyan) to highly conserved (burgundy). View onto the tubulin-binding interface (left) or onto the bottom of the TOG domain (right).

Figure S3, related to Figure 2A

Candidates identified as putative Cep104 (proximity) interactors in BioID and cross-linking experiments.

A) Top, Coomassie stained SDS-PAGE gel showing the elutions from a Cep104 pull-down (employing site-specific biotinylation of Avitag-WT BirA tagged Cep104 in vivo) in the presence or absence of cross-linking. The gel corresponds to ~10% of the material that was subjected to mass-spectrometric analysis. Bottom, Proteins identified by mass-spectroscopy that showed a ten-fold or higher enrichment of exclusive unique peptide counts compared to the control. **B)** Top, Coomassie stained SDS-PAGE gel showing ~10% of the elution from a Cep104 BioID experiment (biotinylation of proteins in the vicinity of BirA* tagged Cep104 in vivo). The remainder of the elution was subjected to mass-spectrometric analysis. Bottom, proteins with a ten-fold or higher enrichment of exclusive unique peptide counts compared to the control.

Figure S4, related to Figure 2B

The Cep104 ZNF domain is necessary for the Cep104-Nek1 interaction in vivo.

Representative fluorescence micrographs of a re-routing and capture assay (Wong and

Munro, 2014) in U2OS cells with Nek1 and Cep104 constructs. N-terminally mCherry-tagged Cep104 or Cep104ΔZNF (Cep104$^{\Delta 746\text{-}880}$), fused at their C-terminus to the HA tag and the outer-mitochondrial membrane targeting transmembrane domain of monoamine oxidase (MAO) were co-transfected with GFP-Nek1 into U2OS cells, fixed after 24 hours and visualized using a confocal microscope. A mitochondrial stain (MitoTracker Deep-red, ThermoFisher Scientific) and DAPI were used to mark mitochondria and DNA respectively. Please note that ectopically expressed GFP-Nek1 reroutes only to the mitochondrially anchored Cep104 when Cep104's ZNF domain is present. Scale bars, 5 μm.

Figure S5, related to Figure 3A

Binding of CP110$^{906\text{-}936}$ and Nek1 to the Cep104 ZNF array is mutually exclusive. Right: Western blot showing a pull-down experiment with GST-Cep104 ZNF beads and lysates from Hek293 cells transiently overexpressing 3xFlag-tagged human Nek1 in the presence of an excess of recombinant CP110$^{906\text{-}936}$ or *Danio rerio* STIL$^{404\text{-}448}$. Please note that *Danio rerio* STIL$^{404\text{-}448}$ did not transfer/Ponceau-stain well as revealed by the Coomassie-stained SDS-PAGE gel of the input material shown on the left.

Figure S6, related to Figure 4

Multiple sequence alignment and structural comparison of Cep104's ZNF domain.

A) Marked with coloured lines and circles are the zinc-coordinating residues and the residues that constitute the three hydrophobic clusters that mediate the packing of the individual ZNFs into a globular domain. Indicated in red is residue S763 that was mutated (S763E) to increase solubility of the ZNF domain. **B)** The globular arrangement of the Cep104 ZNF array is novel. Comparison of the Cep104 ZNF domain with a similar ZNF array from TRAF6 (ribbon presentation, pdb 3hcs).

Supplementary References

Wong, M., and Munro, S. (2014). Membrane trafficking. The specificity of vesicle traffic to the Golgi is encoded in the golgin coiled-coil proteins. Science (New York, NY) *346*, 1256898.

Figure S1, related to Figure 1A

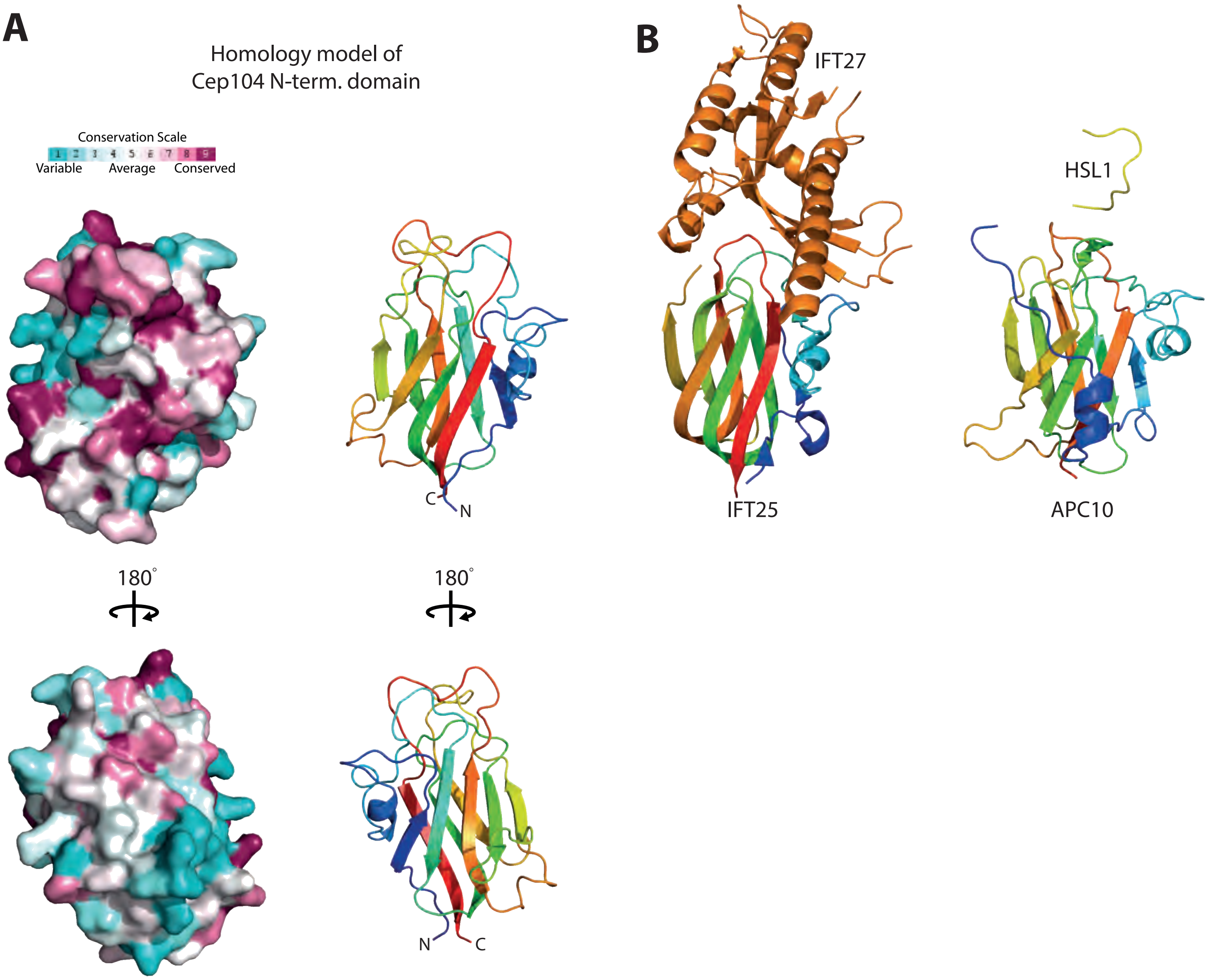

Figure S2, related to Figure 1B-D

A

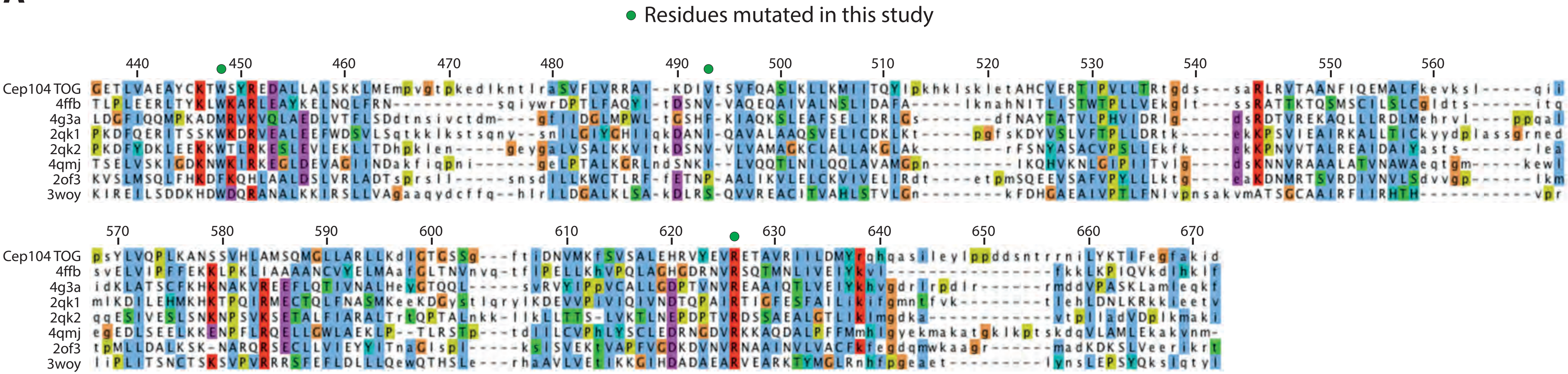

Figure S2, related to Figure 1B-D

B

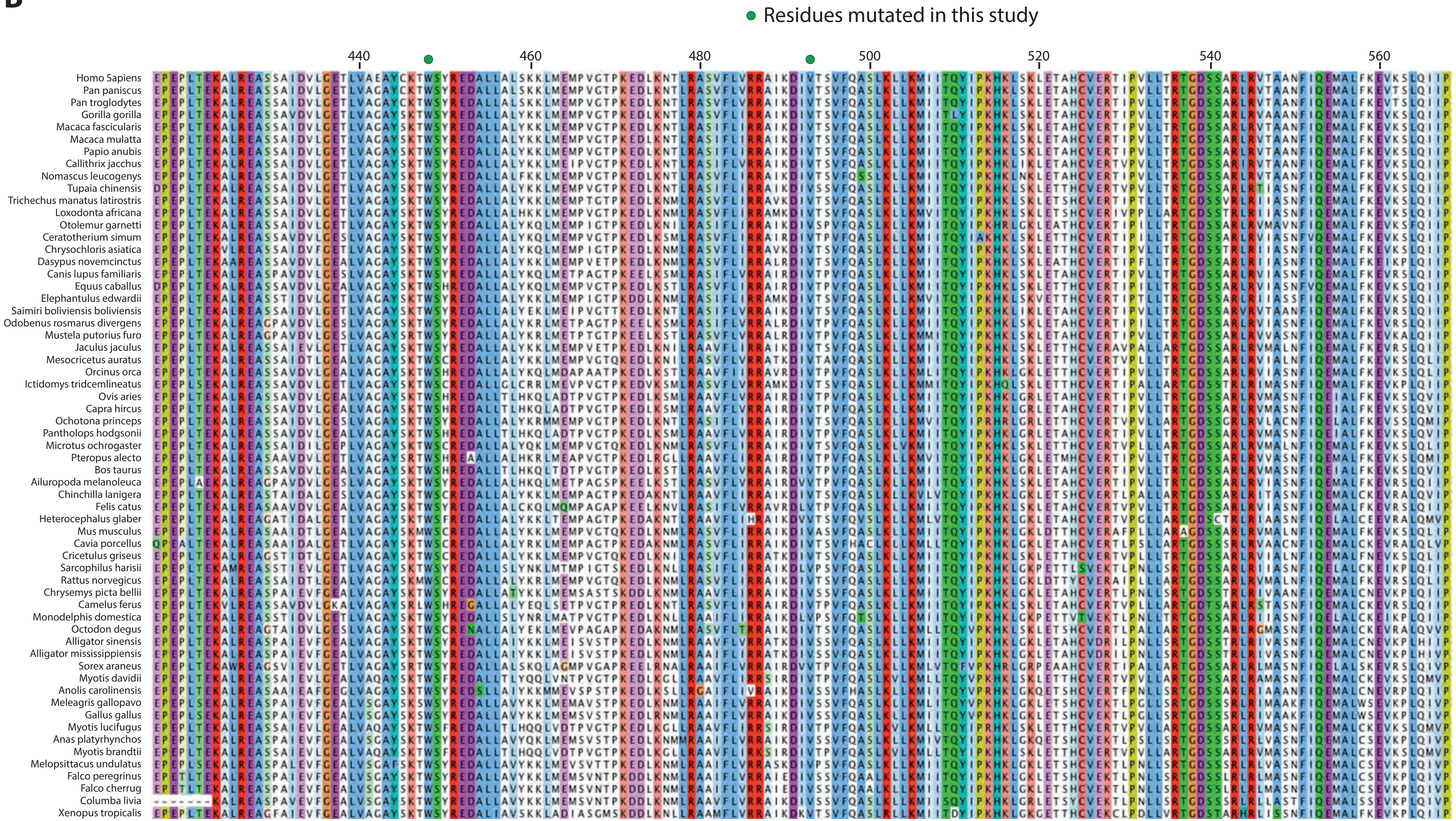

Figure S2, related to Figure 1B-D

B (continued)

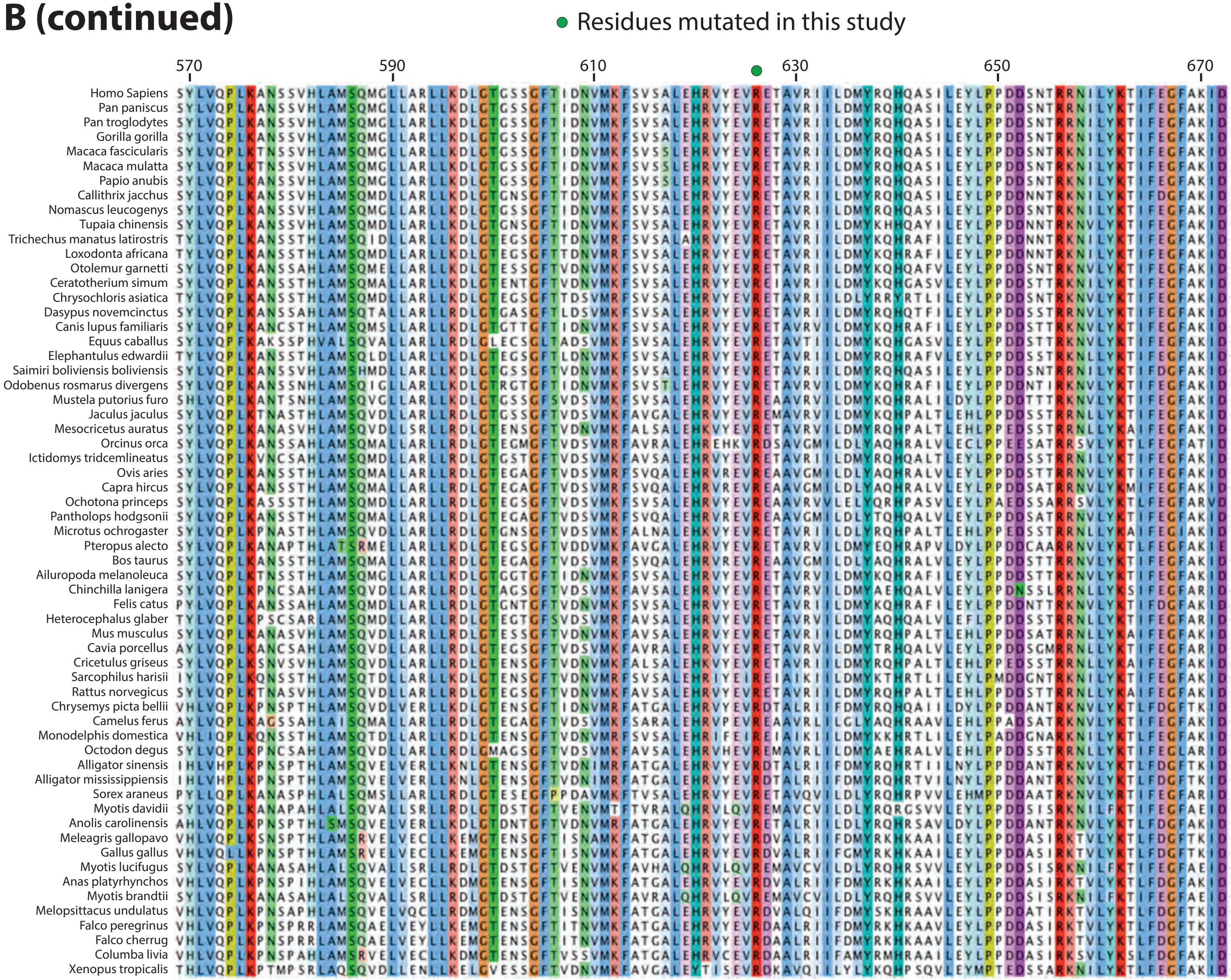

Figure S2, related to Figure 1B-D

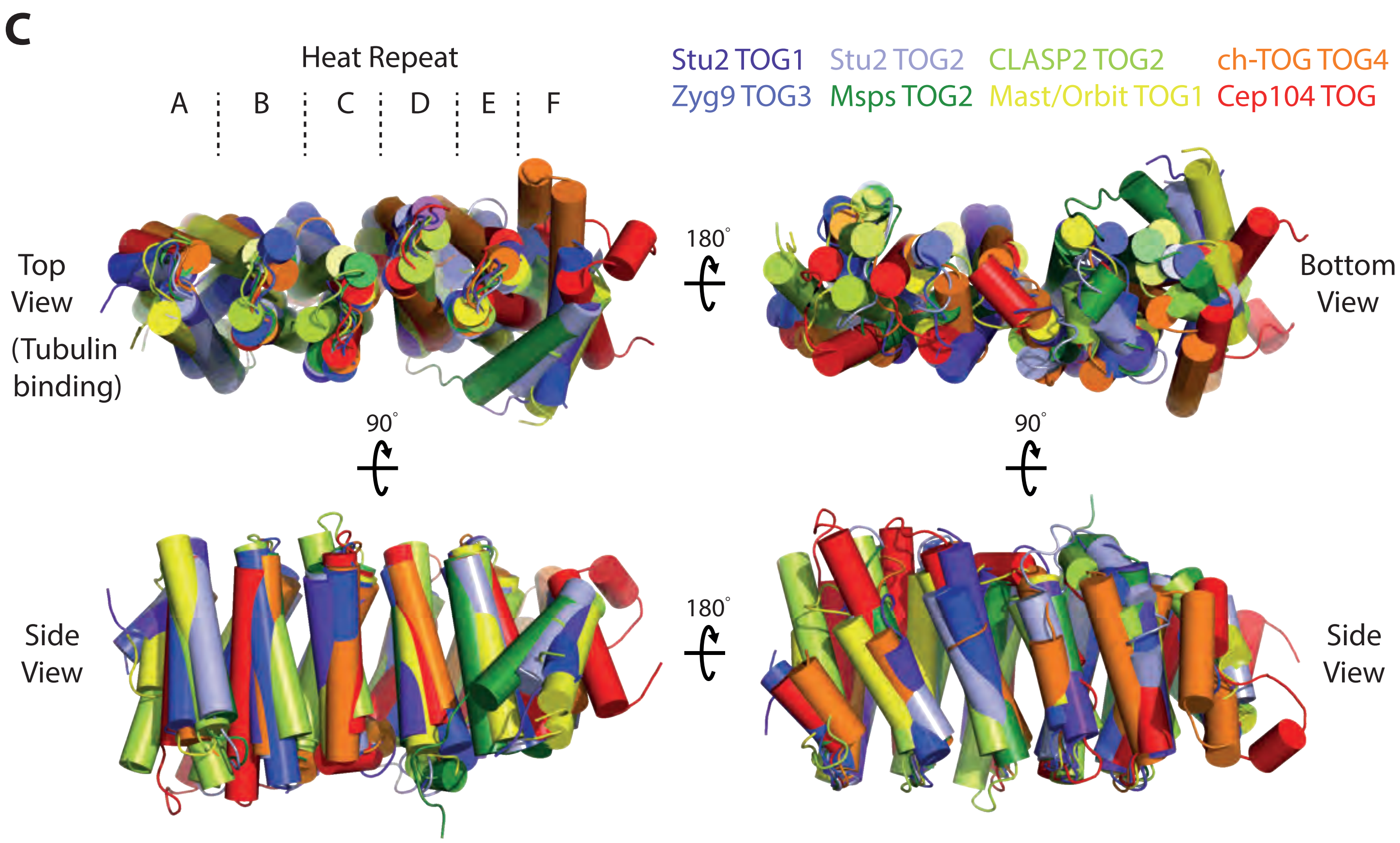

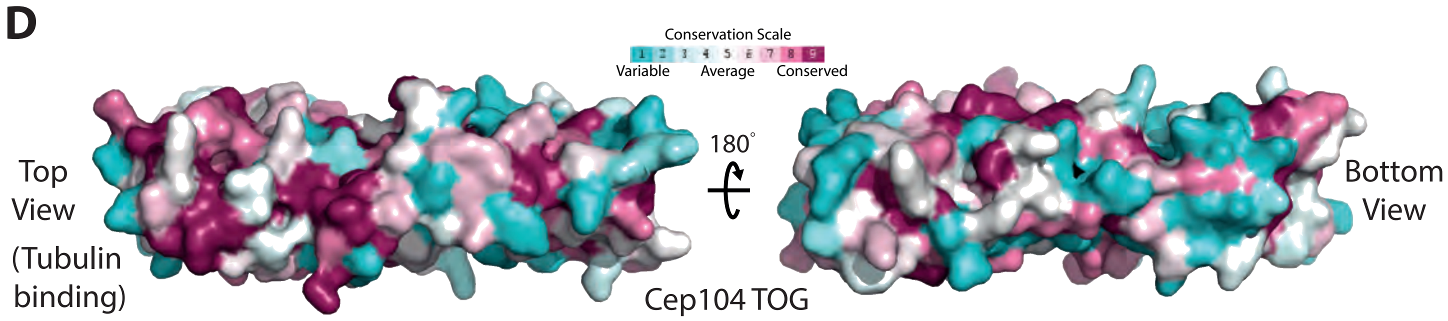

Figure S3, related to Figure 2A

A

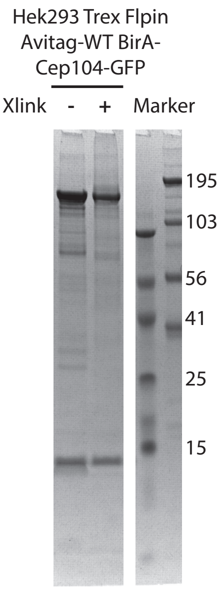

B

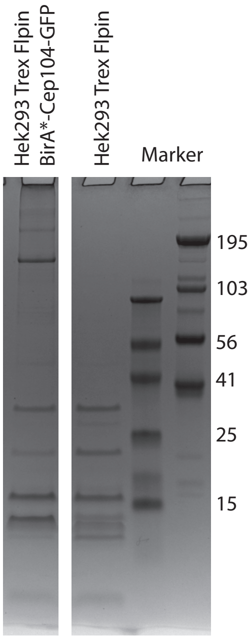

Xlinking

PCCB
LMNB1
MDH2
RBMX
RUVBL2
CLTC
MYH10
NCL
DNAJA2
B4E1G2
lmna
SFPQ
TPR
CCT2
ACTN4
EIF4G1
SPTAN1
B2R7C5
B2R8R5
Cep290
CSE1L
EIF3A
VCP
HUWE1
TNPO1
Nek1

BioID

HMPREF9541_01744	NUDC	lacZ	TCHP	NEDD1	SLK	PPP6R3
CEP104	PDLIM1	UBAP2L	ATXN2L	ZYX	TJP2	SRP68
FLNA	HSP90AB1	NUFIP2	TUBB	PA2G4	SMG7	PSMC2
CCT8	MAP4	Q53GZ6_HUMAN	PCBP1	EIF4G2	TRIM9	NOTCH2
AHNAK	EIF5	SNAP29	CAMSAP1	SPAG9	RASSF8	DBN1
PCM1	VIM	KIAA1009	DPYSL3	CSDE1	XIAP	SCIN
CEP170	AZI1	RANBP3	GEMIN5	RPAP3	CCDC66	SEC16A
CD2AP	MYH9	GPATCH1	B3KY28_HUMAN	PIBF1	IFT74	PSMD12
Q53HG7_HUMAN	NUP214	ANKRD17	SUGT1	TACC1	EEF2	CEP290
CCP110	RANGAP1	RUVBL1	CLINT1	TMF1	FKBP4	TCP1
CEP350	MRE11A	DDX3X	EPB41	DST	SYAP1	KTN1
EIF4G1	MPHOSPH9	DPYSL2	TNRC6B	RANBP2	CCT2	
MKL2	CSPP1	UBAP2	NSFL1C	HNRPK	EPB41L2	
TAGLN2	MAP7D3	DBNL	PKM	CCDC85C	RAI14	
HSPA1B	SKA3	KIAA1671	WWOX	EIF2A	B0FTY2_HUMAN	
ANKHD1	HAUS6	TP53BP2	A8KAL2_HUMAN	GOLGB1	KIAA1524	
CRKL	MAP1B	IRAK1	ANXA2	PLS3	ZC3H15	
LUZP1	TLN1	EIF4B	CKAP2	ENO1	WDR44	
LARP1	NEK1	HDLBP	DLGAP5	STIP1	B4DTX5_HUMAN	
SERBP1	TJP1	IRS4	DNAJB1	PXN	FAM21A	
CRK	ALMS1	ERC1	KNS2	VCL	EIF4ENIF1	
CEP97	CORO1B	CALD1	CENPH	SPAG5	EPS15	
PRRC2C	CNN3	C1orf198	BRAP	PPP1R12A	DNAJC7	
EPS15L1	DVL2	STAU1	CKAP5	KIF5B	REPS1	
MLLT4	EFHD2	VCPIP1	USP15	SNX1	CLMN	
OFD1	PDLIM5	PRRC2A	ABLIM1	ERBB2IP	SLAIN2	

Hits with ≥ 10-fold enrichment of exclusive, unique peptide counts compared to control

Figure S4, related to Figure 2B

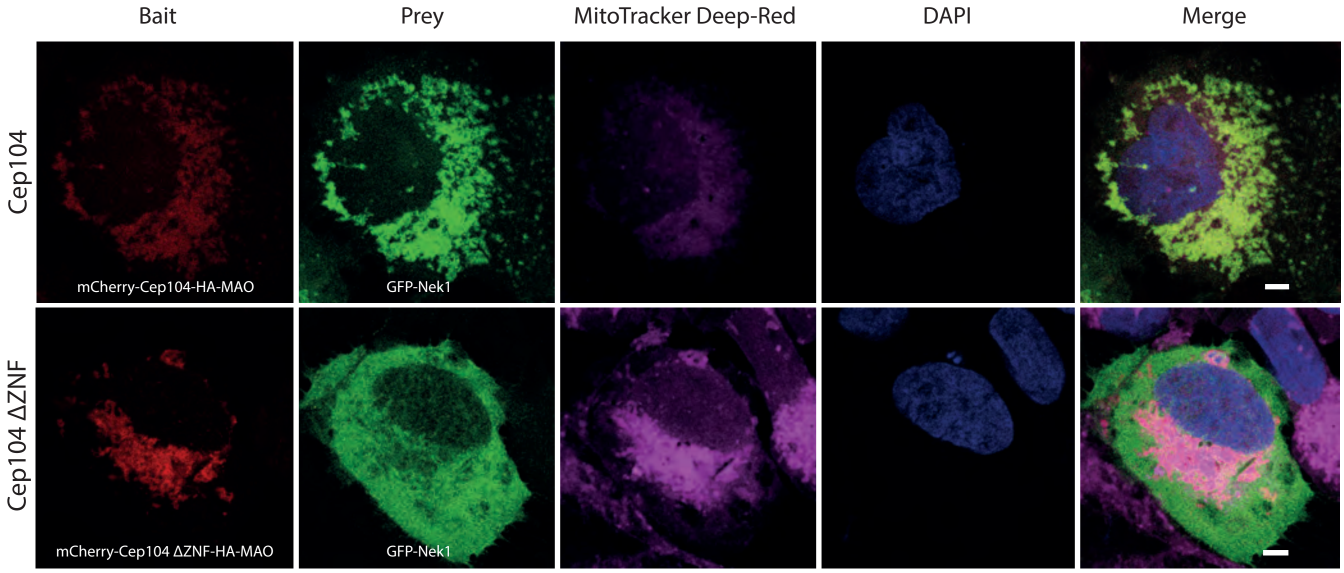

Figure S5, related to Figure 3A

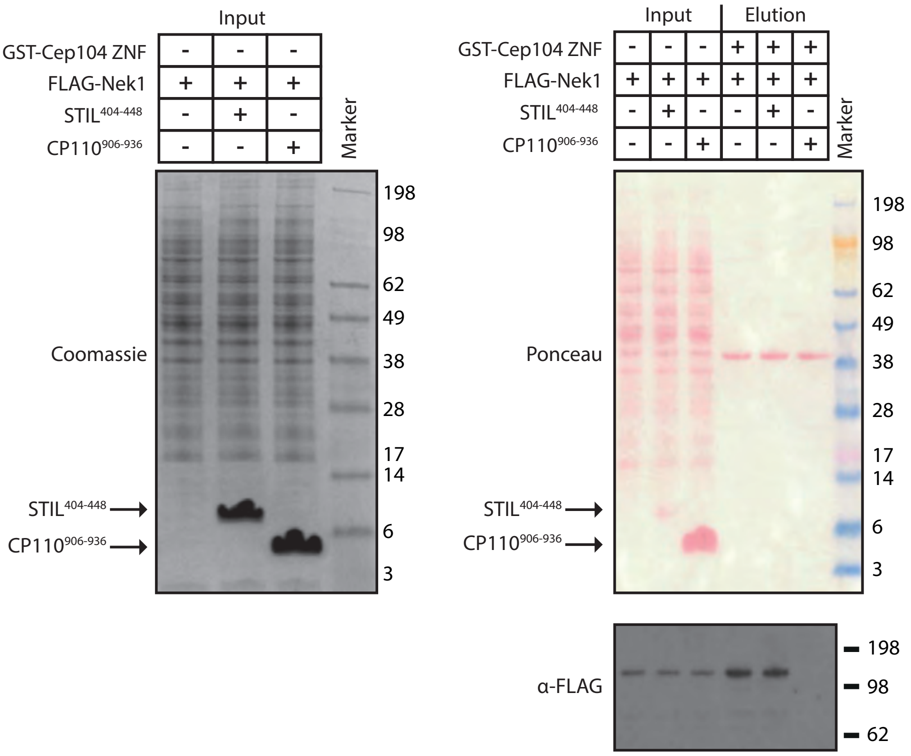

Figure S6, related to Figure 4

A

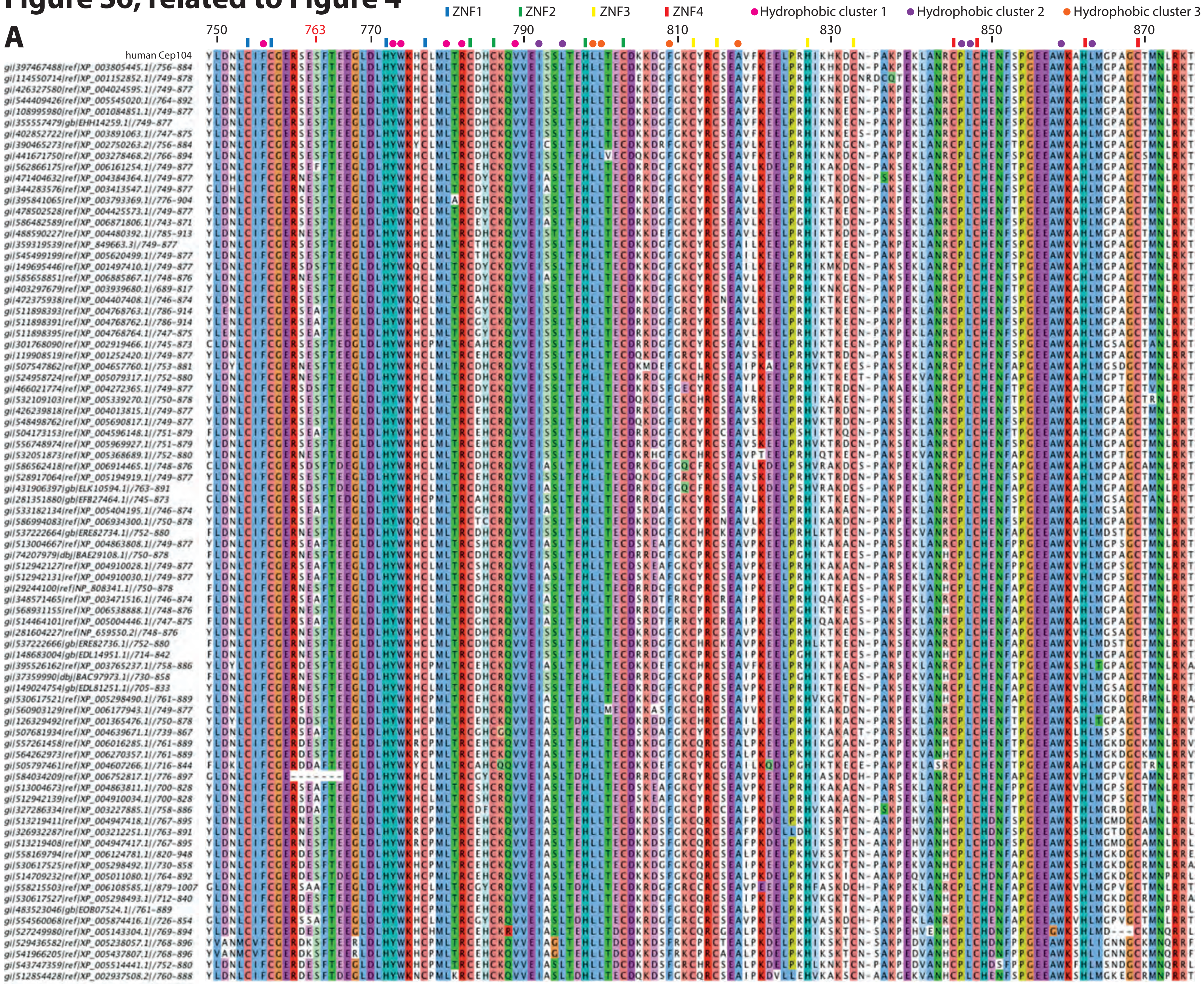

Figure S6, related to Figure 4

B

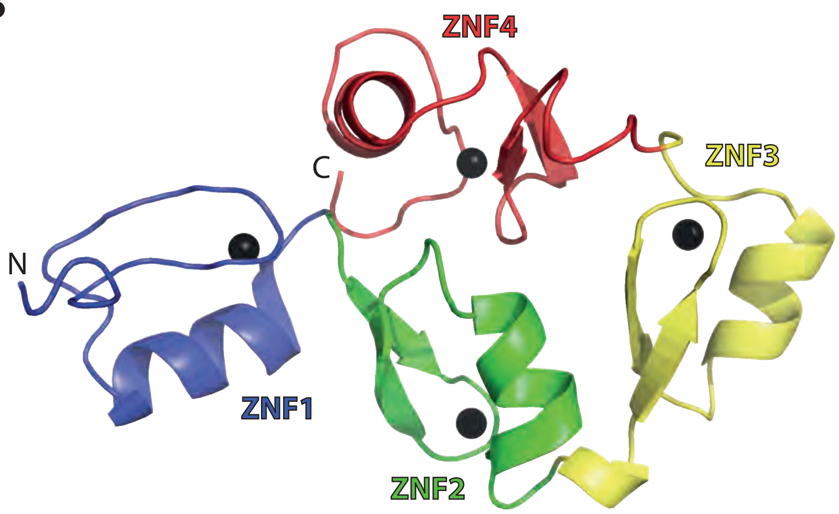

Cep104 ZNF array

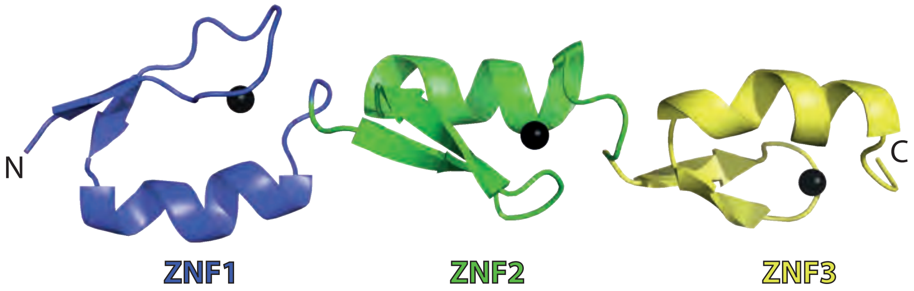

TRAF6 ZNF array
